# Elevated peripheral and nervous system inflammation is associated with decreased short-chain fatty acid levels in Zika virus-infected macaques

**DOI:** 10.1128/jvi.01003-25

**Published:** 2025-08-19

**Authors:** Ty A. Schroeder, Jennifer A. Manuzak, Charlene J. Miller, Andrew T. Gustin, Christopher M. Basting, Ryan K. Cheu, Adrian Velez, Connor B. Driscoll, Jennifer Tisoncik-Go, Luca Schifanella, Tiffany Hensley-McBain, Claudya A. Evandy, Elise A. Smith, Debbie Bratt, Robin Shields-Cutler, Jeremy Smedley, Deborah H. Fuller, Dan H. Barouch, Megan A. O'Connor, Michael Gale, Nichole R. Klatt

**Affiliations:** 1 Department of Surgery, Division of Surgical Outcomes and Precision Medicine Research, University of Minnesota 5635, Minneapolis, Minnesota, USA; 2 Washington National Primate Research Center, University of Washington 7284, Seattle, Washington, USA; 3 Department of Pharmaceutics, University of Washington 7284, Seattle, Washington, USA; 4 Division of Immunology, Tulane National Primate Research Center, Tulane University 5783, Covington, Louisiana, USA; 5 Department of Immunology, University of Washington 7284, Seattle, Washington, USA; 6 Center for Innate Immunity and Immune Disease, University of Washington 7284, Seattle, Washington, USA; 7 McLaughlin Research Institute 88978, Great Falls, Montana, USA; 8 Department of Biology, Macalester College 7569, Saint Paul, Minnesota, USA; 9 Oregon National Primate Research Center, Oregon Health and Sciences University 6684, Hillsboro, Oregon, USA; 10 Department of Microbiology, University of Washington 7284, Seattle, Washington, USA; 11 Center for Virology and Vaccine Research, Beth Israel Deaconess Medical Center, Harvard Medical School 1811 , Boston, Massachusetts, USA; The Ohio State University, Columbus, Ohio, USA

**Keywords:** Zika virus, microbiome, cerebrospinal fluid, central nervous system, short-chain fatty acids, neopterin, tryptophan catabolism, kynurenine pathway, serotonin, inflammation

## Abstract

Zika virus (ZIKV) infection typically results in mild symptoms; however, it can cause elevated inflammation in the CNS and the development of more serious conditions, including severe neurological syndromes. Previous studies have demonstrated that ZIKV infection may cause mucosal dysfunction as well as elevated inflammation in rectal and vaginal mucosal tissue. Here, we used non-human primate (NHP) models and clinical specimens from ZIKV-infected humans to examine microbiome, inflammation, and microbial translocation in the gut, blood, and CNS. We observed elevated markers indicative of microbial translocation and inflammation in the CNS of ZIKV-infected macaques and humans. While overall taxonomic shifts in microbial communities did not differ, bacterial-derived short-chain fatty acid (SCFA) levels were reduced. Decreased SCFA levels significantly negatively correlated with elevated peripheral and CNS inflammatory markers, suggesting a link between ZIKV-driven disease pathology and microbial function. Taken together, our study provides new insight into a previously unconsidered mechanism underlying ZIKV pathogenesis.

**IMPORTANCE:**

Zika virus poses a significant burden on public health, demonstrated by the 2015–2016 epidemic that remains with devastating health consequences especially in countries such as Brazil. However, the mechanisms that underlie what leads to inflammation of the central and peripheral nervous system and disease in Zika virus have been poorly characterized. Here, we demonstrated that Zika virus resulted in gut microbiome dysbiosis, which led to decreased short-chain fatty acids, increased inflammation, and microbial translocation, likely brought on by reduced gut barrier integrity. This study represents the first evidence that microbial dysbiosis and gut damage may underlie Zika virus pathogenesis and ensuing neurological effects, providing a potential target for intervention for future virus pandemics.

## INTRODUCTION

As has been acutely evident by the COVID-19 pandemic, viral infections are a major health problem, and understanding viral-induced effects on human health is critical. Zika virus (ZIKV) is an arbovirus of the *Flaviviridae* family that threatens the health of millions globally and was the cause of a major epidemic in 2015–2016. ZIKV infection typically results in mild symptoms including rash, fever, and conjunctivitis in adults, but it has occasionally been linked to more severe conditions, including Guillain-Barré syndrome (GBS) and Zika congenital syndrome (1). Previous work assessing a Brazilian cohort of ZIKV-infected human adults demonstrated an increased incidence of serious neurological syndromes in infected individuals, such as demyelinating GBS, axonal GBS, encephalitis, and transverse myelitis (2). Serologic studies have detected Zika viral infections among humans globally, most recently in the Americas and Caribbean (3).

ZIKV has been detected in central nervous system (CNS) tissue (2, 4–8), which likely causes ZIKV-associated neuropathy and neuroinflammation (6, 9–11). However, additional mechanisms may drive CNS inflammation in the context of ZIKV infection. For example, inflammatory monocyte and macrophage accumulation in the brain has been observed during viral infections, including other Flavivirus infections like West Nile virus (12) and Japanese encephalitis virus (13). Accumulation of activated monocytes/macrophages in the CNS during ZIKV infection could result in elevated production of neopterin, a biomarker of immune activation produced by monocytes and macrophages in response to interferon stimulation (14, 15). Indeed, elevated neopterin levels have been observed in the cerebrospinal fluid (CSF) of individuals with viral or bacterial CNS infections (16). Additionally, monocyte activation via IFN-γ stimulation or recognition of pathogen-associated molecular patterns (PAMPs) via pattern recognition receptors (PRRs) could induce the gene expression and enzymatic activity of indoleamine 2,3-dioxygenase 1 (IDO-1), the first enzyme involved in the kynurenine pathway of tryptophan catabolism (17–20). Altered tryptophan catabolism and kynurenine levels have been observed in many different viral infections (18), as well as in CNS disorders such as AIDS-dementia complex and Huntington’s disease (21–23). Thus, innate immune cell inflammatory and metabolic responses could contribute to CNS inflammation in the context of ZIKV infection. However, the impact of ZIKV infection on these processes and their role in ZIKV pathogenesis has not yet been investigated.

In addition to the detrimental effects of ZIKV infection in the CNS, previous work has shown that ZIKV can replicate in mucosal compartments, including the intestinal and vaginal mucosa (24, 25). Elevated intestinal mucosal dysfunction due to ZIKV infection (24) could potentiate systemic and eventually CNS inflammation. For example, disruptions in mucosal barrier integrity during ZIKV infection could allow for microbial translocation from the lumen of the gastrointestinal (GI) tract into the periphery and CNS. Increased exposure to these translocated microbial products could drive CNS inflammation, potentially by inducing neopterin production or tryptophan catabolism in monocytes. Additionally, GI microbial dysbiosis, defined as imbalances in microbial communities, has been observed in chronic viral infections, including HIV (26–28) and viral hepatitis (29). As the intestinal microbiome plays an important role in the maintenance of intestinal homeostasis (30), shifts in microbial taxonomy or functionality subsequent to ZIKV infection may provide a potential mechanism for elevated microbial translocation, increased systemic inflammation, and risk for negative neurological outcomes. However, no studies to date have investigated the occurrence and potential links between ZIKV-induced mucosal disruptions, microbial translocation and dysbiosis, and peripheral and CNS inflammation.

Macaque models of HIV, influenza, Ebola virus, and other pathogens display infection kinetics and pathology like human infections, enabling the detailed study of these diseases (31). Given the difficulty in identifying and sampling acute ZIKV infection in humans, the macaque model is similarly invaluable for studying the earliest stages of ZIKV pathogenesis. Recent studies in rhesus macaques have detected ZIKV persistence in multiple tissues as late as 35 days post-infection (dpi) (32). Additionally, ZIKV infection elicits a robust immune response in rhesus macaques that includes ZIKV-specific T cell and neutralizing antibody (Nab) responses that confer protection against reinfection (33). Finally, our group has previously shown that ZIKV infection in pigtail macaques results in a rapid innate immune response in the periphery and recruitment of innate immune cells to the intestinal mucosa (24).

Given the limited knowledge regarding whether ZIKV-associated mucosal dysfunction could drive CNS inflammation and overall ZIKV pathogenesis, here, we sought to evaluate the link between intestinal mucosal alterations and peripheral and CNS immune disruption in the context of ZIKV infection. To do this, we used the pigtail macaque (PTM) model to evaluate associations between the level of microbial translocation and systemic and CNS innate immune activation and metabolic activity during ZIKV infection. Additionally, we explored ZIKV-induced alterations in intestinal microbial community structure and function as a potential mechanism underlying systemic and CNS inflammation. Finally, to support our PTM findings, we performed similar assessments using samples from ZIKV-infected rhesus macaques (RM) and humans. Samples, including stool, vaginal and rectal swabs, CSF, and peripheral blood, were collected from RM, and human serum was obtained from the World Reference Collection for Emerging Viruses and Arboviruses.

## RESULTS

### Infection and sampling of macaques with ZIKV

Pigtailed macaques (PTMs; *n* = 8) were infected with the Brazilian Zika isolate (Brazil_2015_MG, GenBank: KX811222.1). ZIKV inoculation and sample collection were completed in two separate, non-overlapping cohorts of four animals each (cohort 1: *n* = 4 female PTMs; cohort 2: *n* = 4 male PTMs). ZIKV inoculum dose was based on the feeding behavior of the mosquito vector, *Aedes aegypti*, which deposits virus multiple times during skin probing prior to accessing a capillary for acquisition of the blood meal (34). Animals did not exhibit overt clinical symptoms subsequent to ZIKV inoculation. However, we observed that anemia occurred in the females, which could also be associated with menses (Fig. S1). Additionally, macroscopic mucosal shedding was observed in the gastrointestinal tract during endoscopic procedures post-infection. Clinical signs data are not shown. As previously demonstrated, PTMs exhibited plasma viremia by 3 dpi, but viral loads dropped to undetectable levels by 7 dpi (24). Blood, stool, rectal swabs, and cerebral spinal fluid (CSF) were collected pre- and post-infection with ZIKV. The data presented in the current study were generated from cryopreserved samples after all sample collection for both cohorts had been completed. Of note, gastrointestinal (GI) sampling (rectum and colon biopsies collected via endoscopy) was also performed in this PTM study, and data generated using these tissues have been previously published (24).

In addition, plasma and CSF samples were collected from ZIKV-infected rhesus macaques (RMs) enrolled in a previously completed study (4) and were used in a collaborative effort to supplement findings in the PTM model. The RMs were infected with a lower viral dose of the Brazil/AKV2015 isolate and, as previously shown, exhibited lower initial viral loads that were measurable until 14 dpi (4).

### Increased markers of inflammation in CSF and plasma during ZIKV infection

**Fig 1:**
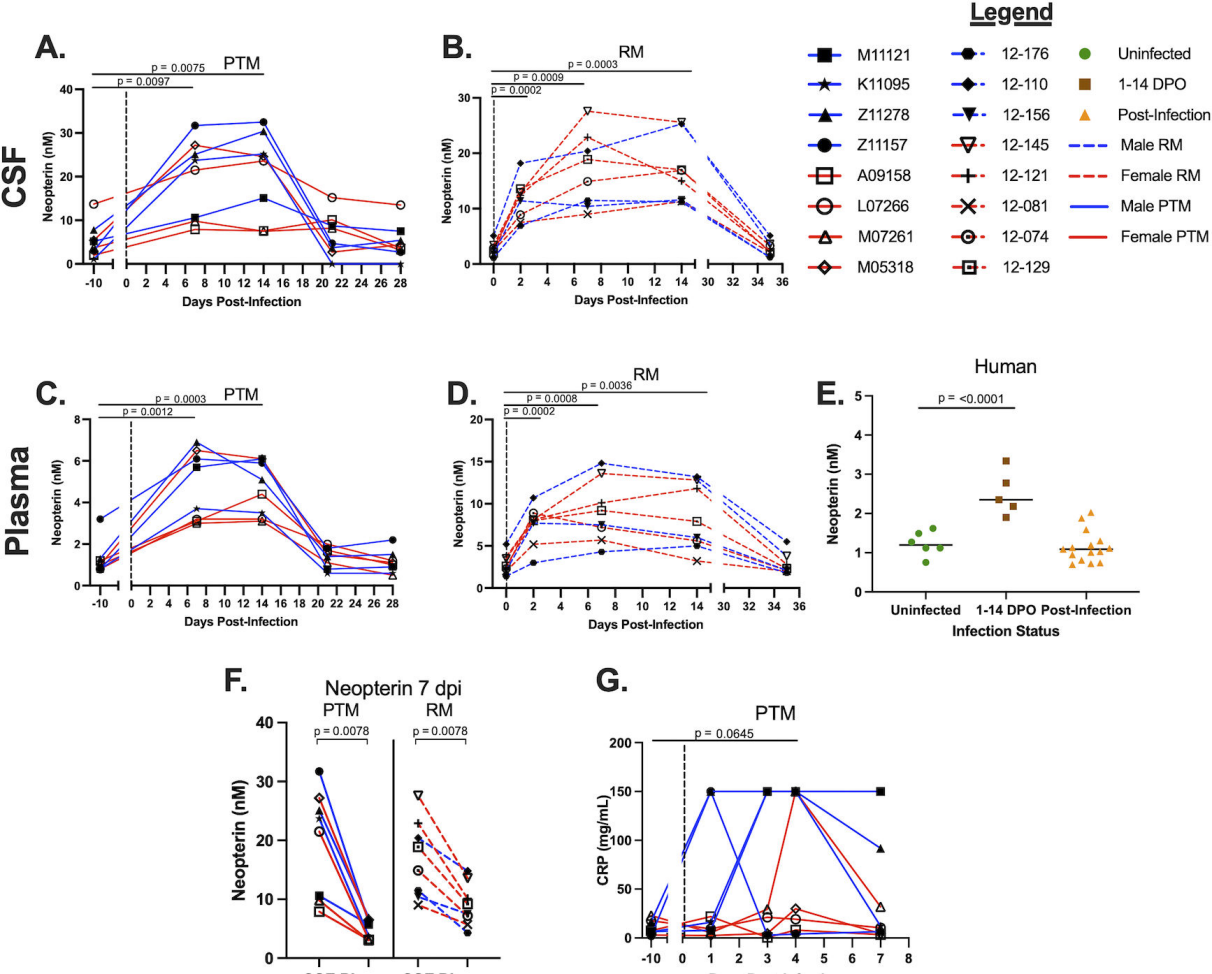
Increased levels of peripheral and CNS inflammatory markers during ZIKV infection. Liquid chromatography tandem mass spectrometry (LC-MS/MS) was used to detect neopterin concentrations in the CSF of PTMs and RMs and in the plasma of PTMs, RMs, and humans throughout ZIKV infection. Additionally, CRP was measured in the plasma of PTMs during acute ZIKV infection by ELISA. (A–E) Neopterin concentrations in the CSF of PTMs (A) and RMs (B), plasma of PTMs (C) and RMs (D), and serum of humans (E) infected with ZIKV. (F) Neopterin concentrations in CSF were compared to those in plasma for both PTMs and RMs. (G) Plasma levels of CRP were assessed in ZIKV-infected PTMs. In panels A–D and F and G, each animal is represented by a different symbol. Solid or dashed lines connect data from the same animal. Solid blue lines indicate male PTMs; solid red lines indicate female PTMs; dashed blue lines indicate male RMs; and dashed red lines indicate female RMs. Vertical dotted lines indicate the time point at which animals were inoculated with ZIKV. Statistical significance between the pre-infection baseline and post-ZIKV infection time points was calculated using a repeated measures ANOVA, with the Geisser-Greenhouse correction and a Dunnett’s multiple comparisons post-test. In panel E, uninfected humans are indicated by green circles; humans experiencing ZIKV symptoms from 1 to 14 days post-onset (DPO) of ZIKV symptoms are indicated by brown squares; and humans post-ZIKV infection (15–156 DPO) are indicated by orange triangles. Black bars indicate median values. Statistical significance between human sample time points was evaluated using a one-way ANOVA with a Dunnett’s multiple comparisons post-test. In all plots, horizontal bars with *P*-values indicate time points that were statistically significant from each other.

Neopterin is produced by monocytes and macrophages in response to IFN-γ stimulation (14, 15) and is commonly used as a diagnostic marker for generalized immune activation in the context of a wide variety of infections, including bacterial, viral, and parasitic diseases (14). Elevated levels of neopterin have been observed in the CNS of individuals in various conditions, including HIV infection (35) and CNS lymphoma (36). To determine whether ZIKV infection resulted in elevated neopterin levels in the CNS, we quantified CSF levels of neopterin in ZIKV-infected PTMs and RMs. In PTMs, a significant increase of neopterin was detected in CSF at 7 dpi, which was sustained until 14 dpi (*P* = 0.0097 and 0.0075, respectively), returning to baseline by 21 dpi (Fig. 1A). In the CSF of RMs, a significant increase in neopterin was detected as early as 2 dpi (*P* = 0.0002), which was maintained at 7 dpi and 14 dpi (*P* = 0.0009 and 0.0003, respectively), before returning to baseline by 35 dpi (Fig. 1B).

We next evaluated whether ZIKV infection resulted in elevated neopterin production in the periphery of PTMs and RMs, and we supplemented these observations with assessments of peripheral neopterin levels in ZIKV-infected humans, using serum obtained from the Global Virus Network (GVN) Zika serum bank. In PTMs, a significant increase in neopterin was detected at 7 dpi and remained elevated at 14 dpi (*P* = 0.0012 and 0.0003, respectively), returning to baseline by 21 dpi (Fig. 1C). In the plasma of RMs, a significant increase of neopterin was detected as early as 2 dpi (*P* = 0.0002) and remained elevated at 7 and 14 dpi (*P* = 0.0008 and 0.0036, respectively), before returning to baseline at 35 dpi (Fig. 1D). In humans, a significant increase in neopterin was detected between uninfected individuals and 1–14 DPO of ZIKV (*P* < 0.0001; Fig. 1E), indicating an upregulation of neopterin within the first two weeks of ZIKV infection, in agreement with our NHP results. Interestingly, the magnitude of neopterin formation was significantly higher in the CSF compared to the plasma at 7 dpi (*P* = 0.0078 for both PTMs and RMs), possibly indicating increased localized inflammatory responses (Fig. 1F).

In order to assess the overall level of peripheral inflammation within the first week of ZIKV infection, we characterized plasma levels of C-reactive protein (CRP), an acute-phase protein that has been used as an indicator of inflammatory responses (37). We observed that plasma levels of CRP increased by 4 dpi compared to baseline, although this difference did not reach statistical significance (*P* = 0.0645; Fig. 1G). Taken together, these data indicate that ZIKV infection of PTMs, RMs, and humans results in elevated systemic and CNS inflammation.

### Tryptophan catabolism is increased in plasma and CSF throughout ZIKV infection

**Fig 2:**
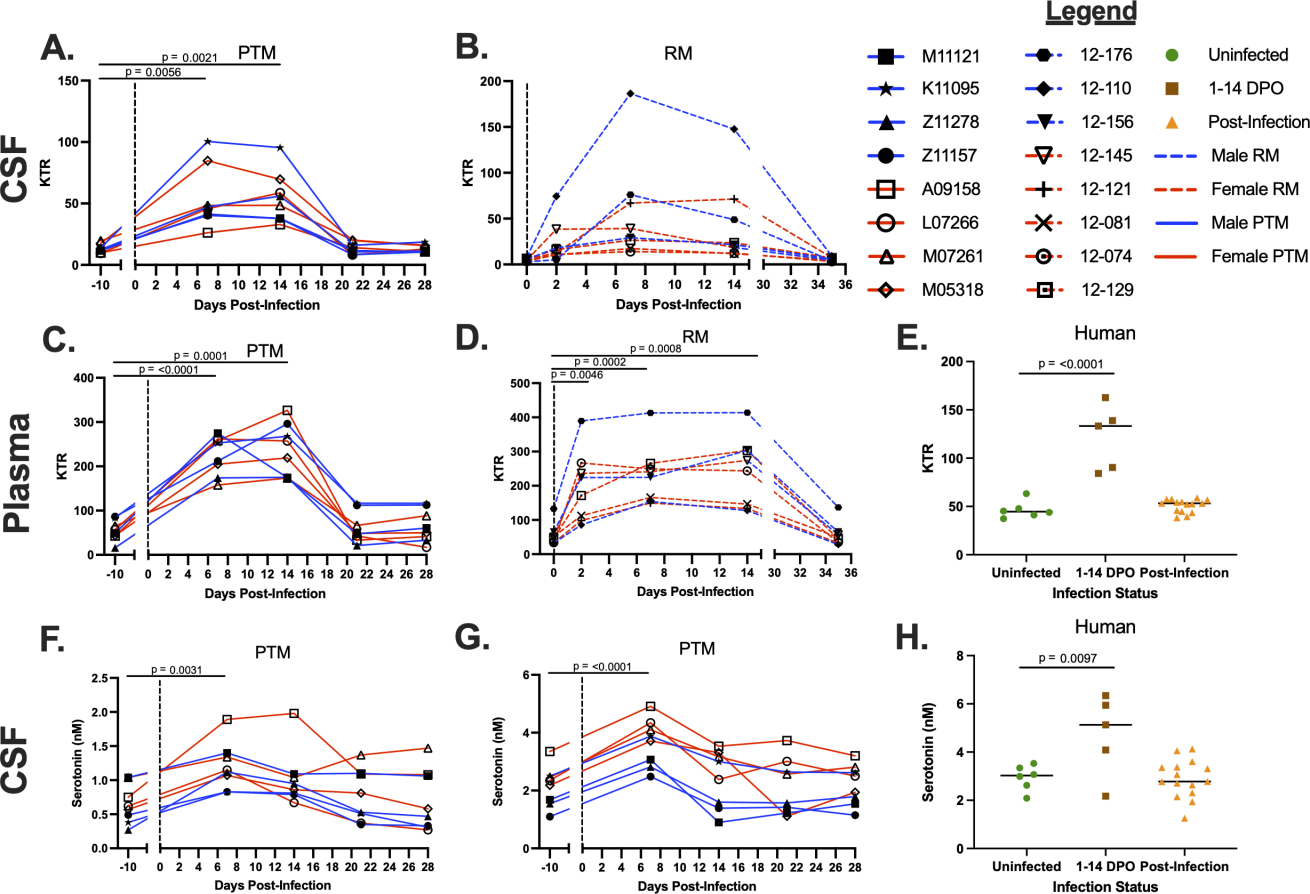
Increased kynurenine:tryptophan ratio (KTR) and serotonin levels are observed during ZIKV infection. Liquid chromatography tandem mass spectrometry (LC-MS/MS) was used to detect kynurenine and tryptophan concentrations in the CSF of PTMs and RMs and in the plasma of PTMs, RMs, and humans throughout ZIKV infection. The KTR was calculated, and the quotient was multiplied by 1,000. Additionally, serotonin was measured in the plasma and CSF of PTM during ZIKV infection by LC-MS/MS. (**A–E**) KTR in the CSF of PTMs (**A**) and RMs (**B**), plasma of PTMs (**C**) and RMs (**D**), and serum of humans (**E**) infected with ZIKV. (**F–H**) Serotonin concentrations in the CSF of PTMs (**F**), plasma of PTMs (**G**), and serum of humans (**H**) infected with ZIKV. In panels A–D and F–G, each animal is represented by a different symbol. Solid or dashed lines connect data from the same animal. Solid blue lines indicate male PTMs; solid red lines indicate female PTMs; dashed blue lines indicate male RMs; and dashed red lines indicate female RMs. Vertical dotted lines indicate the time point at which animals were inoculated with ZIKV. Statistical significance between the pre-infection baseline and post-ZIKV infection time points was calculated using a repeated measures ANOVA, with the Geisser-Greenhouse correction and a Dunnett’s multiple comparisons post-test. In panels E and H, uninfected humans are indicated by green circles, humans experiencing ZIKV symptoms from 1 to 14 days post-onset (DPO) of ZIKV symptoms are indicated by brown squares, and humans post-ZIKV infection (15–156 DPO) are indicated by orange triangles. Black bars indicate median values. Statistical significance between human sample time points was evaluated using a one-way ANOVA with a Dunnett’s multiple comparisons post-test. In all plots, horizontal bars with *P*-values indicate time points that were statistically significant from each other.

Previous work has shown that tryptophan metabolism by indoleamine-2,3-dioxygenase (IDO) (17–20) is upregulated during viral infections (18, 38), and significant changes to the kynurenine-tryptophan ratio (KTR) could be due to increased monocyte activation and are associated with microbial dysbiosis (27). Moreover, increased amounts of kynurenine in the CNS have been linked to cognitive disorders and neurological deficits (39). We, therefore, measured the levels of kynurenine (K) and tryptophan (T) in the CSF of ZIKV-infected PTMs and RMs. The KTR was calculated, with a higher ratio indicating increased tryptophan catabolism. We detected a significant increase in the KTR in the CSF of PTM by 7 dpi, and this increase was sustained until 14 dpi (*P* = 0.0056 and 0.0021, respectively) before returning to baseline at 21 dpi (Fig. 2A). Conversely, no differences in the KTR in the CSF of RMs were observed at any post-ZIKV infection time point compared to baseline (Fig. 2B).

To determine if the kynurenine pathway of tryptophan metabolism is upregulated in the periphery during ZIKV infection, we assessed the KTR in the plasma of ZIKV-infected PTMs, RMs, and humans. A significant increase in the KTR was detected in the plasma of the PTMs at 7 dpi (*P* ≤ 0.0001) and was sustained until 14 dpi (*P* = 0.0001), returning to baseline by 21 dpi (Fig. 2C). Similarly, a significant increase in the KTR was observed in the plasma of the RMs as early as 2 dpi (*P* = 0.0046) and remained significant at 7 and 14 dpi (*P* = 0.0002 and 0.0008, respectively), before returning to baseline at 35 dpi (Fig. 2D). Additionally, a significant increase in KTR was observed in ZIKV-infected humans at 1–14 DPO of symptoms compared to uninfected individuals (*P* ≤ 0.0001; Fig. 2E), in agreement with our NHP results.

While production of kynurenine accounts for approximately 90% of tryptophan metabolism, approximately 3% is used for the synthesis of serotonin (5-HT) (40). Given our findings that ZIKV infection results in alteration of the KTR, we next investigated whether serotonin levels differed in the plasma and CSF of PTMs and in the plasma of humans. Serotonin levels were not measured in RMs due to lack of sample availability. A significant increase in serotonin levels was detected in the CSF of PTMs by 7 dpi (*P* = 0.0031) and was elevated at 14 dpi, although this increase was not statistically significant (Fig. 2F). Serotonin levels in the CSF of PTMs returned to baseline by 21 dpi (Fig. 2F). Further, serotonin levels in the plasma of PTMs significantly increased at 7 dpi (*P* ≤ 0.0001) and returned to baseline by 14 dpi (Fig. 2G). In human plasma, a significant increase in serotonin levels was seen between uninfected individuals and individuals at 1–14 DPO with ZIKV infection (*P* = 0.0097; Fig. 2H). Together, these data reveal that both tryptophan metabolism pathways become overactivated during the first week of ZIKV infection, which could be a possible mechanism involved in ZIKV pathogenesis and neurological complications that occur due to infection.

### Microbial translocation may contribute to elevated sCD14 in CSF during ZIKV infection

**Fig 3:**
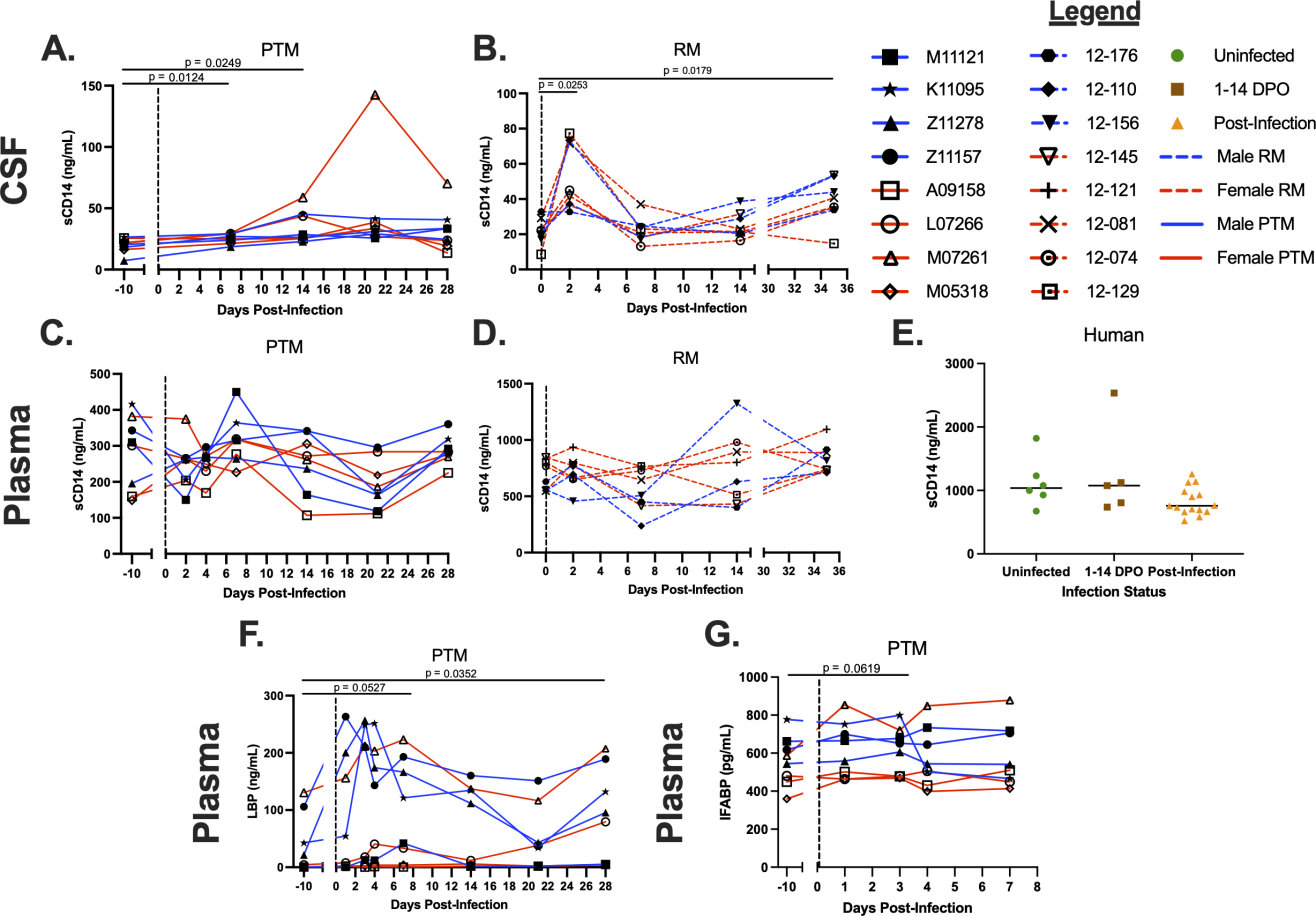
Increased levels of soluble markers of monocyte activation, microbial translocation, and intestinal barrier dysfunction during ZIKV infection. Enzyme-linked immunosorbent assays (ELISAs) were used to detect soluble CD14 (sCD14), lipopolysaccharide binding protein (LBP), and intestinal fatty acid binding protein (I-FABP) in the CSF of PTMs and RMs and in the plasma of PTMs, RMs, and humans throughout ZIKV infection. (**A–D**) sCD14 in the CSF of PTMs (**A**) and RMs (**B**), plasma of PTMs (**C**) and RMs (**D**), and serum of humans (**E**) infected with ZIKV. (**F**) LBP levels in the plasma of PTM. (**G**) I-FABP levels in the plasma of PTM. In panels A–D and F and G, each animal is represented by a different symbol. Solid or dashed lines connect data from the same animal. Solid blue lines indicate male PTMs; solid red lines indicate female PTMs; dashed blue lines indicate male RMs; and dashed red lines indicate female RMs. Vertical dotted lines indicate the time point at which animals were inoculated with ZIKV. Statistical significance between the pre-infection baseline and post-ZIKV infection time points was calculated using a repeated measures ANOVA, with the Geisser-Greenhouse correction and a Dunnett’s multiple comparisons post-test. In panel E, uninfected humans are indicated by green circles, humans experiencing ZIKV symptoms from 1 to 14 days post-onset (DPO) of ZIKV symptoms are indicated by brown squares, and humans post-ZIKV infection (15–156 DPO) are indicated by orange triangles. Black bars indicate median values. Statistical significance between human sample time points was evaluated using a one-way ANOVA with a Dunnett’s multiple comparisons post-test. In all plots, horizontal bars with *P*-values indicate time points that were statistically significant from each other.

Increased microbial translocation has previously been observed in many viral infections and is consistently associated with systemic inflammation and co-morbidities (28, 41–45). We hypothesized that microbial translocation during ZIKV infection could contribute to increased monocyte activation and result in inflammatory responses. Cluster of differentiation 14 (CD14) is a co-receptor for the bacterial product LPS; it is expressed either as a glycosylphosphatidylinositol (GPI)-anchored membrane protein on monocytes or macrophages (46), or as a soluble protein (sCD14) that is produced via secretion or enzymatic cleavage from the cell surface (47). sCD14 is considered a marker of monocyte activation due to microbial exposure (48), and translocated microbial products that enter the nervous system may induce sCD14 expression in this compartment (49). We, thus, measured sCD14 concentrations in the CSF throughout ZIKV infection of PTM and RM. A significant increase in sCD14 was detected in the CSF at 7 dpi in the PTMs and was sustained through 14 dpi (*P* = 0.0124 and 0.0249, respectively; Fig. 3A). In the RMs, a significant increase in sCD14 was detected in the CSF as early as 2 dpi (*P* = 0.0253; Fig. 3B), despite no detectable ZIKV RNA in the CSF at that time point (4). CSF sCD14 levels declined slightly at 7 and 14 dpi in RMs but increased significantly at 35 dpi (*P* = 0.0179; Fig. 3B). We also examined sCD14 in the periphery but detected no significant changes in plasma sCD14 levels in either PTMs or RMs throughout ZIKV infection (Fig. 3C and D, respectively). We also assessed serum CD14 levels in ZIKV-infected humans and observed no significant differences in serum CD14 levels in uninfected individuals compared to 1–14 days post-onset (DPO) and post-infection with ZIKV (Fig. 3E).

Peripheral levels of lipopolysaccharide (LPS)-binding protein (LBP), an acute-phase protein that aids in binding of LPS to PRRs, have been used previously as a surrogate marker of microbial translocation (50–52). Thus, we assessed plasma levels of LBP before and after ZIKV infection of PTMs. We observed a trend towards increased levels of LBP in plasma at 7 dpi (*P* = 0.0527; Fig. 3F). Plasma levels of LBP remained elevated and became significantly increased at 28 dpi (*P* = 0.0352; Fig. 3F).

Given the trend towards increased LBP in plasma and significant increases in sCD14 in the CSF, we next assessed the integrity of the mucosal epithelial barrier. Intestinal fatty acid-binding protein (I-FABP) is a cytoplasmic protein found in intestine epithelial cells (4). I-FABP is released into the periphery after enterocyte disruption and thus has been used as a soluble biomarker of intestinal epithelial cell damage (53). To determine whether ZIKV infection caused intestinal epithelial barrier disruption, we evaluated plasma levels of I-FABP in ZIKV-infected PTMs during acute infection. Levels of I-FABP in the plasma during ZIKV infection remained consistent with the levels observed prior to ZIKV infection, although we did observe a trend towards increased I-FABP at 3 dpi (*P* = 0.0619; Fig. 3G). To further investigate a shift in gut barrier integrity, circulating zonulin concentrations were measured in PTM plasma. However, there was no detectable zonulin in the plasma samples. Taken together, our findings suggest that microbial product translocation into the periphery may contribute to increased CNS inflammation in ZIKV infection of PTMs, RMs, and humans, although further assessment of CNS is needed to determine how microbial translocation directly impacts CNS inflammation.

### Microbial taxonomy in the gastrointestinal tract of PTMs during ZIKV infection

**Fig 4:**
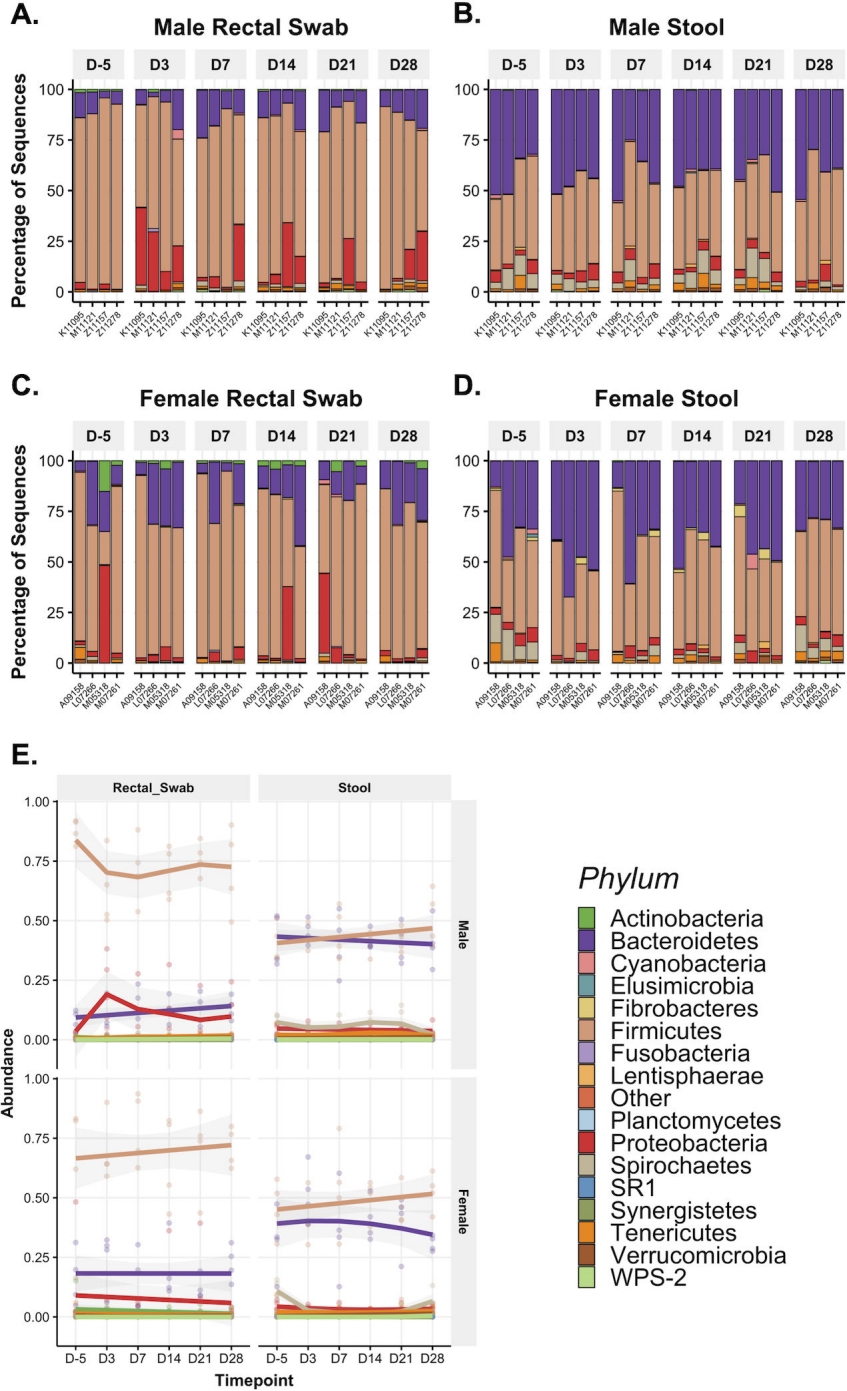
Bacterial community composition at the phyla level in the rectum and stool of PTMs before and after ZIKV infection. 16S rRNA gene sequencing was used to characterize microbial phyla in stool and rectal swabs collected from PTM prior to and throughout ZIKV infection. (**A–D**) Relative abundance taxonomic plots of microbial phyla in male PTM rectal swabs (**A**), male PTM stool (**B**), female PTM rectal swabs (**C**), and female PTM stool (**D**). Vertical colored bars represent the percentage of total sequences for specific phyla in individual animals prior to ZIKV infection and at each time point post-ZIKV infection. (**E**) Smoothed mean relative abundance of bacterial phyla in each of the indicated sample types in male and female PTM. Solid colored lines represent the mean abundance for specific bacterial phyla. Gray shading overlaying each colored line represents standard error bounds. Matched colored dots surrounding each colored line represent the specific abundances of each bacterial phyla for individual animals.

Shifts in the GI microbiota occur frequently in the context of disease or infection (i.e., microbial dysbiosis), and such dysbiosis has been shown to alter mucosal integrity, peripheral PAMP levels, and local immune responses (27, 28, 54–58). West Nile virus, a close relative of ZIKV, has previously been shown to promote significant GI alterations (59). To determine if ZIKV infection impacts the intestinal microbiome, we compared the relative abundance of rectal and stool microbiota of PTMs before and after ZIKV infection. Prior to infection, both rectal and stool microbiomes in male and female PTMs were dominated by bacteria in the Firmicutes phyla, followed by Bacteroidetes and Proteobacteria (Fig. 4A through D). An increase of Proteobacteria was detected at 3 dpi in male rectal swabs, and levels remained elevated above baseline for the duration of the study (Fig. 4A and E). This relative increase was reciprocated by a relative decrease in Firmicutes (Fig. 4A and E). No shifts were detected post-infection at the phylum level in female rectal swabs (Fig. 4C and E). Microbial phyla in male stool appeared relatively stable following infection (Fig. 4B and E). Spirochaetes levels in female stool decreased at 3 dpi and remained below baseline throughout the study (Fig. 4D and E). In addition to phyla level assessments, we also evaluated genus level changes in microbial communities throughout ZIKV infection. We observed a transient non-significant increase in *Prevotella* in stool from female PTMs at 3 dpi, which decreased below baseline abundances by 28 dpi (Fig. S1). Additionally, there was a transient non-significant increase in *Streptococcus* in rectal swabs from female PTMs at 7 dpi, which returned to baseline abundances by 21 dpi (Fig. S1). No other shifts in microbial abundance were detected post-infection at the genus level in male and female rectal swabs and stool (Fig. S1).

**Fig 5:**
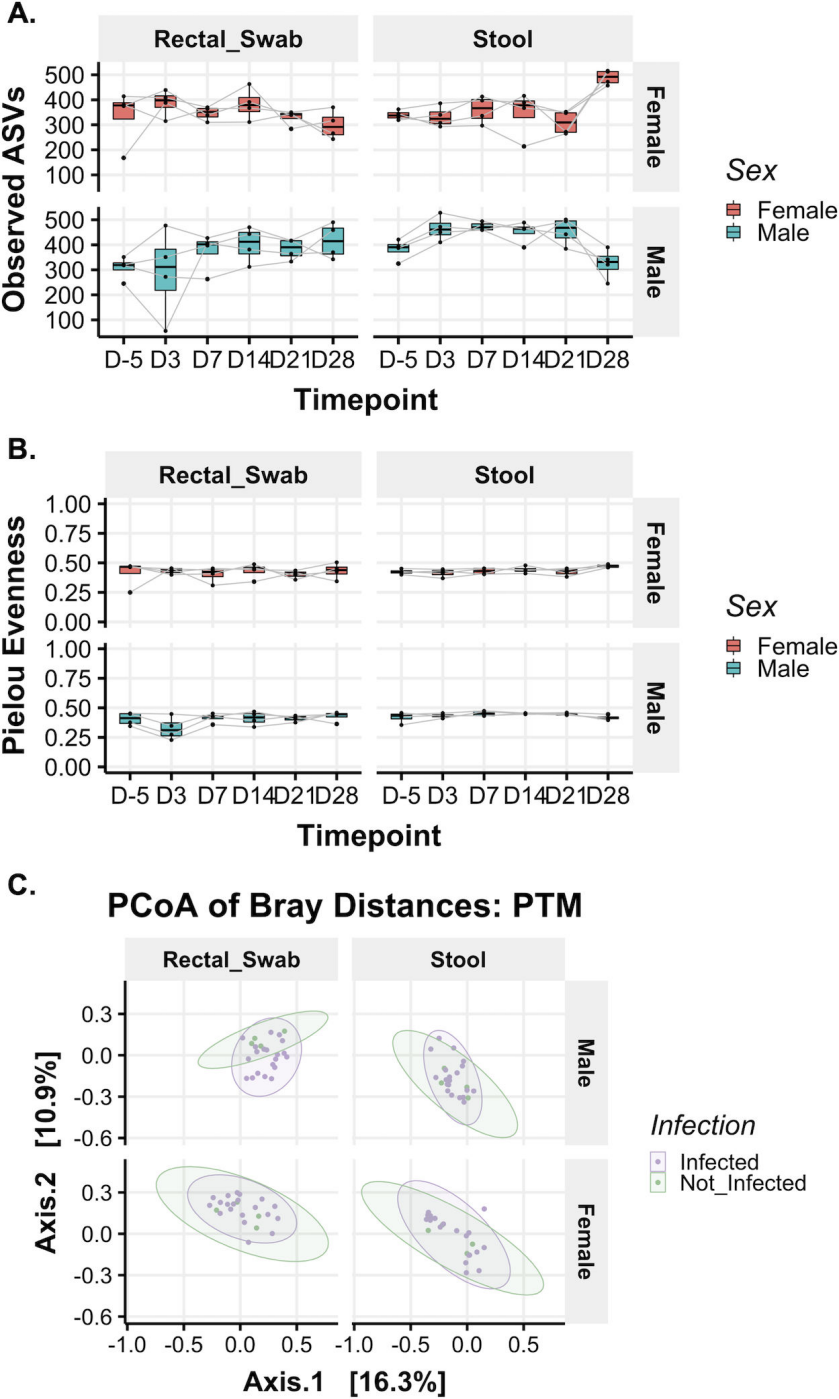
Similar bacterial community richness and evenness throughout ZIKV infection of PTMs. Measures of alpha-diversity were used to examine overall changes in stool and rectal bacterial community structure prior to and throughout ZIKV infection of PTMs. (**A and B**) Bacterial community richness (**A**) and evenness (**B**) in rectal swabs and stool from female (pink) and male (teal) PTMs. Box and whisker bars represent 25–75 percentile and minimum and maximum number of observed amplicon sequence variants. Horizontal bars within each box represent the median. Black dots connected by gray lines that overlay box and whisker plots represent the total number of observed ASVs for individual animals at each time point. (**C**) Principal components analysis of Bray-Curtis beta-diversity in male and female PTM rectal swabs and stool prior to and after ZIKV infection. Pre-ZIKV data points are shown in green, and post-ZIKV data points are shown in purple. Shaded ovals for each group represent data ellipses.

When considering the impact of ZIKV infection on microbiome structure, we did not observe significant changes in community richness or evenness (Fig. 5A and B). Microbial diversity trends were not influenced by environmental variables, such as origin of colony or paired caging (Table S1); however, we did note a significant difference in microbiota richness between male and female PTMs in stool samples (Fig. S2). Analysis of microbiota composition, as assessed through Bray-Curtis beta-diversity analysis, demonstrated that microbial composition clustered by sex independent of infection status, suggesting that the strongest differences in overall microbial composition were associated with sex (Fig. 5C). When segregated by sex, baseline samples fell within post-infection clusters for both rectal swabs and stool (Fig. 5C). However, given that the male and female studies were performed at different times, cohort-specific changes cannot be ruled out. We did note a small number of amplicon sequence variants (ASVs) that experienced statistically significant log_2_-fold shifts from baseline following infection (Fig. S3). However, these findings must be considered in the context of a single baseline observation, making it unclear if the changes observed were due to ZIKV-specific disease processes or were within the expected range of variation for a one-month span of time. In sum, these data indicate that ZIKV infection induced only subtle shifts in microbial taxonomy in the rectum and stool of male and female PTMs.

### Depletion of short-chain fatty acids (SCFAs) in stool of PTMs during ZIKV infection

**Fig 6:**
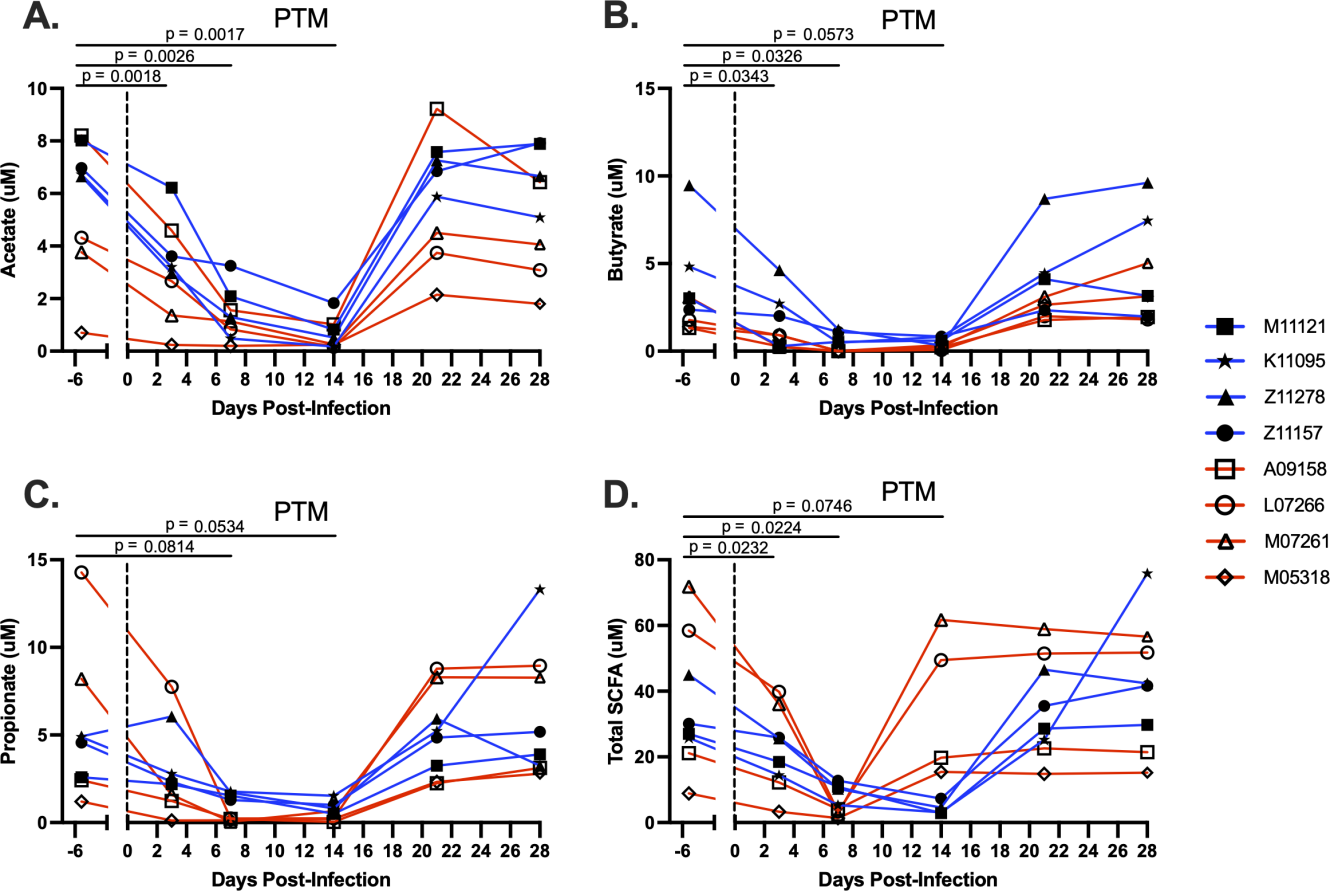
SCFAs are depleted in stool of PTMs during ZIKV infection. Gas chromatography mass spectrometry (GC-MS) was used to detect concentrations of short-chain fatty acids (SCFAs) in the stool of PTM throughout ZIKV infection. Concentrations of (**A**) acetate, (**B**) butyrate, (**C**) propionate, and (**D**) total short-chain fatty acids (SCFA) from stool were evaluated. Total SCFAs included concentrations of acetate, butyrate, propionate, iso-butyrate, valerate, and iso-valerate. Each animal is represented by a different symbol. Solid lines connect data from the same animal. Solid blue lines indicate male PTMs; solid red lines indicate female PTMs. Vertical dotted lines indicate the time point at which animals were inoculated with ZIKV. Statistical significance between the pre-infection baseline and post-ZIKV infection time points was calculated using a repeated measures ANOVA, with the Geisser-Greenhouse correction and a Dunnett’s multiple comparisons post-test. In all plots, horizontal bars with *P*-values indicate time points that were statistically significant from each other.

The intestinal microbiome contributes to GI health through multiple mechanisms, including the fermentation of metabolic products and the production of short-chain fatty acids (SCFAs), which play a role in regulating immunity and inflammation (60, 61). Although we did not observe significant alterations in microbial taxonomy in ZIKV-infected PTMs, it is possible that subtle changes in community structure may still impact microbial functionality. Therefore, to evaluate intestinal microbial community function, we measured SCFA levels in the stool of ZIKV-infected PTMs. Acetate, butyrate, and propionate, which are the primary end products of the fermentation process (62), were individually measured by GC-MS. Total SCFA production was also measured by GC-MS, which additionally includes measurements of iso-butyrate, valerate, and iso-valerate. Concentrations of acetate in the stool pre-infection ranged from 0.70 μM to 8.20 μM and significantly decreased in all animals by 3 dpi (*P* = 0.0018; Fig. 6A). This decrease was sustained through 7 and 14 dpi (*P* = 0.0026 and 0.0071, respectively) before returning to baseline at 21 dpi (Fig. 6A). Butyrate concentrations pre-infection ranged from 1.33 μM to 9.47 μM and significantly decreased in all animals by 3 dpi (*P* = 0.0343). As with acetate levels, this decrease was sustained through 7 and 14 dpi (*P* = 0.0326 and 0.0573, respectively) before returning to baseline at 21 dpi (Fig. 6B). Concentrations of propionate in the stool pre-infection ranged from 1.20 μM to 14.28 μM. Although levels were decreased by 7 dpi through 14 dpi compared to baseline, this decrease did not reach statistical significance (*P* = 0.0813 and 0.0534, respectively; Fig. 6C). Levels of propionate returned to baseline at 21 dpi (Fig. 6C). Additionally, total SCFA production significantly decreased at 3 dpi through 7 dpi (*P* = 0.0232 and 0.0224, respectively; Fig. 6D). Interestingly, female PTMs began recovering total SCFA levels by 14 dpi, while male PTMs sustained total SCFA depletion through 14 dpi (Fig. 6D). Taken together, our findings indicate that although the overall microbial community structure did not appear to be altered during ZIKV infection in PTMs, ZIKV may induce functional changes resulting in altered levels of microbial-derived metabolic products.

### Associations between increased inflammatory biomarkers and indicators of microbial functionality during ZIKV infection

**Fig 7:**
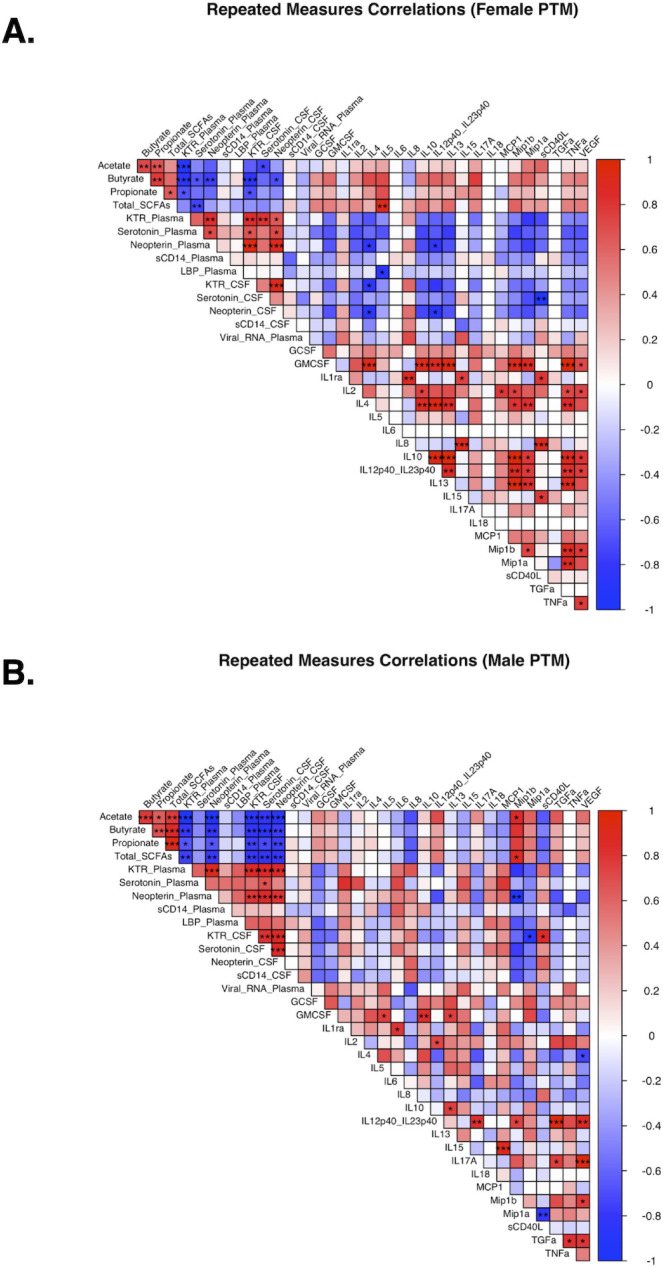
Microbial translocation markers correlate with plasma and CSF markers. Viral RNA (copies/mL), SCFAs from stool, and biomarkers were assessed across time points in PTM using repeated measures correlations. Trends in relationships are presented by sex with (**A**) female and (**B**) male PTM correlation matrices. Correlation results from rmcorr (*r*_rm_) are represented in the correlation matrix through color, where −1 is dark blue, while 1 is dark red for each comparison; correlation strength near 0 or at 0 is white. Significant thresholds for FDR adjusted *P*-values (*Q*) included *** for < 0.001, ** for 0.001 < *q* < 0.01, and * for 0.01 < *q* < 0.05.

Previous studies have suggested that altered SCFA production may provide a mechanism by which microbial disruptions could influence peripheral and CNS inflammatory pathway production (63–67). To investigate if changes in SCFA levels were associated with changes in immunological parameters in ZIKV-infected PTMs, we performed repeated-measures correlations between SCFAs, markers of inflammation in plasma and CSF, viral RNA (copies/mL), and markers of microbial translocation (Fig. 7). Analyses were separated based on male or female PTM study groups, and data from all time points were used. We observed that in female PTMs, SCFAs were negatively correlated with plasma and CSF KTR (Fig. 7A). These negative correlations reached statistical significance between the SCFAs butyrate and propionate in the plasma, and all three SCFAs measured in the CSF (Fig. 7A). SCFAs were negatively associated with serotonin levels, reaching statistical significance in the plasma with butyrate and serotonin in the CSF with acetate (Fig. 7A). Similarly, SCFAs were negatively associated with neopterin levels, although this only reached statistical significance in the plasma with butyrate (Fig. 7A). Weaker negative associations were observed between all SCFAs and plasma and CSF sCD14, as well as plasma LBP (Fig. 7A).

Similar trends were observed in male PTMs (Fig. 7B). Specifically, SCFAs were negatively correlated with plasma and CSF KTR, and these associations reached statistical significance for acetate, butyrate, propionate, and total SCFAs in both plasma and CSF (Fig. 7B). Plasma and CSF neopterin levels were also negatively correlated with SCFA levels (Fig. 7B). These negative correlations reach statistical significance between plasma/CSF neopterin and all of the SCFA measurements (Fig. 7B). Plasma and CSF serotonin levels were negatively associated with SCFAs, although plasma correlations did not reach statistical significance, while those in CSF did (Fig. 7B). Weaker negative correlations were observed between SCFAs and plasma and CSF sCD14, as well as plasma LBP (Fig. 7B). There were several trends of note that suggest a connection between microbial translocation and infection. LBP had positive associations with KTR, serotonin, and neopterin in plasma and CSF in males (plasma: *r*_rm_ = 0.63, 0.59, 0.58; CSF: *r*_rm_ = 0.61, 0.64, 0.64), with LBP being negatively associated with SCFAs acetate and butyrate in male PTM (*r*_rm_ = 0.46, 0.43), potentially highlighting overall elevated permeability due to infection. Additionally, LBP and MCP-1 in the plasma had a strong positive association in male PTMs (*r*_rm_ = 0.5) that may suggest a connection between neutrophil and macrophage recruitment with microbial translocation. sCD14 measured in the plasma was positively associated with several pro-inflammatory cytokine immune markers, but strongest with IL-1ra and IL-6 (*r*_rm_ = 0.41, 0.62). Interestingly, LBP in the plasma was significantly, negatively associated with anti-inflammatory IL-5 in female PTMs, potentially highlighting a relationship between elevated LBP/translocation and immune dysregulation since IL-5 decreases after the first few days post-infection, whereas LBP increases (*q *= 0.02, *r*_rm_ = −0.87). Other interactions between cytokines and viral load from these data were explored in a previous publication (24). Altogether, these findings demonstrate that disruption of microbial SCFA production is associated with elevated peripheral and CNS inflammation during ZIKV infection, suggesting that alterations in microbial function may play a role in ZIKV pathogenesis.

## DISCUSSION

The importance of the intestinal mucosal immune system and microbiome in maintaining health and homeostasis is widely recognized. However, the role of the mucosal immune system and microbiome in ZIKV infection, which became a rapid global epidemic (65), has not been well described. Indeed, given the significant associations between proper GI function and CNS homeostasis, it is possible that ZIKV-induced alterations in mucosal function could influence ZIKV pathogenesis in the CNS. Here, we used two separate NHP models, as well as clinical samples from ZIKV-infected humans, to explore whether ZIKV-induced elevations in systemic and CNS inflammation could be linked with increased mucosal dysfunction. We observed that ZIKV infection resulted in moderate elevations of microbial translocation markers in the plasma of PTMs, but significant increases in the CSF of PTMs and RMs. Of note, the three male PTMs with increased plasma LBP were the only animals that had detectable virus in the rectum at 7 dpi (24), suggesting a potential link between productive viral replication in rectal mucosal tissue and elevated intestinal barrier disruption, thus allowing for increased microbial translocation. Previously, we demonstrated that this viral persistence in the rectum may be perpetuated in a sex-dependent manner by elevated pro-inflammatory cytokines, particularly MCP-1, which was significantly higher in male PTMs than in female PTMs by 2 dpi. Although other pro-inflammatory cytokines and chemokines, such as IL-1RA, sCD40L, and IL-15, were increased by 2 dpi, they were not associated with viral persistence (24). MCP-1 was observed here to be positively correlated with LBP, demonstrating a possible mechanism of how microbial translocation may be related to viral dissemination into rectal mucosa via mediation from early enhanced MCP-1 production. Interestingly, we did not detect differences in plasma levels of the soluble marker of intestinal barrier damage, I-FABP, in ZIKV-infected PTMs. However, given the notably short half-life of FABPs in the circulation (approximately 11 min) (68), it is possible that our sample collection time points were insufficient to capture transient changes in I-FABP levels. Further work assessing additional indicators of intestinal barrier damage will be needed to fully elucidate the impact of ZIKV on the mucosal epithelium and determine the potential mechanism underlying the loss of intestinal barrier integrity that may allow elevated microbial translocation during ZIKV infection.

Our study revealed a significant increase in neopterin concentrations in the plasma and CSF of both species of NHPs after ZIKV infection. Interestingly, the concentration of neopterin was significantly higher in the CSF compared to plasma in both NHP species, potentially indicating a localized inflammatory response in the CNS during ZIKV infection. Moreover, our observation of elevated CNS levels of neopterin could indicate that activated monocytes are infiltrating and accumulating in the CNS, thus contributing to CNS pathology during ZIKV infection, as has been suggested in HIV infection (69). Our findings in the NHP model were in agreement with observations made from limited clinical samples, with significantly higher concentrations of neopterin found in the plasma of ZIKV-infected humans. Although we were unable to obtain CSF from ZIKV-infected humans, CSF neopterin concentrations could provide a biomarker or preliminary evidence of CNS inflammation and neurological symptoms in patients infected with ZIKV. Previous work has suggested that plasma neopterin levels are elevated in women with fetal growth restriction (70). It is possible, therefore, that ZIKV-induced increases in neopterin in pregnant women could contribute to the development of ZIKV congenital syndrome. Additional work with more clinical samples will be needed to determine the precise mechanism by which elevated neopterin levels occur in the plasma and CNS during ZIKV infection, and the role this may play in the development of fetal neurological deficits during gestation.

Concurrent with elevated neopterin levels, our study revealed a significant increase in the KTR in the plasma and CSF of ZIKV-infected PTMs and RMs. These findings were in agreement with observations made using clinical samples, with a higher KTR found in the plasma of ZIKV-infected humans. In combination with our observations of elevated plasma and CSF neopterin levels, higher peripheral and CNS KTR in ZIKV infection may indicate elevations in monocyte/macrophage activation, which could drive ZIKV neuroinflammation, similar to what has been observed in HIV neuropathogenesis (69). Additionally, elevated catabolism of tryptophan in the CNS of ZIKV-infected individuals could result in higher levels of quinolinic acid, an end product of tryptophan degradation that has been linked to the development of AIDS dementia complex (71). Further studies are needed to determine how altered tryptophan metabolism during ZIKV infection could contribute to systemic and CNS inflammation and immune activation, thereby driving ZIKV pathogenesis.

In addition to upregulated tryptophan metabolism to kynurenine, our study demonstrated increased serotonin levels in the plasma of ZIKV-infected PTMs and humans, and in the CSF of PTMs. In the brain, serotonin modulates critical neurodevelopmental processes, plays a role in cognitive function, and affects mood in humans (40, 72, 73). A previous murine study suggested that maternal inflammation during pregnancy resulted in overactivation of serotonin synthesis, which contributed to adverse fetal brain development (72). It is therefore possible that maternal inflammatory responses during ZIKV infection could cause elevated serotonin production, which may, in turn, impact fetal neurological development. Additional work will be needed to fully examine the mechanism by which ZIKV-induced inflammation and disruption of metabolic processes, such as tryptophan metabolism into kynurenine and serotonin, may contribute to ZIKV pathogenesis and potentially ZIKV congenital syndrome.

Our assessment of stool and rectal microbial communities by 16S rRNA gene sequencing suggested that intestinal microbial community structure, including richness and evenness, is relatively stable in PTM following ZIKV infection. Although our study did not reveal major significant shifts in microbial taxonomy, we did observe decreased levels of SCFAs in the stool of ZIKV-infected PTMs, suggesting a potential impact of ZIKV infection on intestinal bacterial functionality. SCFAs have distinct physiological roles, including maintenance of gut barrier function, and have been shown to act as an energy substrate for colonocytes (74). Individuals with conditions, such as inflammatory bowel disease, obesity, or viral infections like HIV have been shown to experience reductions in SCFA levels (75–78), which may promote further disruption of mucosal homeostasis. Our data demonstrating that SCFA depletion occurred after ZIKV infection could indicate that ZIKV causes generalized mucosal dysfunction. This alteration in mucosal homeostasis could drive chronic peripheral and CNS inflammation, thereby potentiating ZIKV pathogenesis. Taken together, our work suggests that regardless of taxonomic differences, ZIKV infection may impact microbial functionality, which may provide a mechanism underlying mucosal, systemic, and CNS inflammation.

Our study had several limitations. First, the infections and sampling for the male and female PTM cohorts were performed at different times, so observed sex differences may be driven by the variability associated with performing study timelines at separate times. However, we attempted to minimize sampling bias further by generating all the data presented here together from cryopreserved specimens after sample collection for both cohorts was completed. Second, a limitation of our microbiome analysis is that our baseline sampling included only one pre-infection time point. This limits our ability to establish whether any observed community shifts were due to normal variation or a true biological effect of ZIKV infection. Considering the impact that infectious disease processes have on microbial community composition, future assessments should include extensive baseline sampling to enable statistical assessments of variation pre- and post-infection. Third, a limitation of collecting SCFAs from stool is the volatility of the esters. There is a chance that not all SCFAs were captured during these methods, as these samples were exposed to air for unknown amounts of time prior to collection. Fourth, human clinical samples only included 1–14 DPO or post-infection that had varied time points, while our NHP models had baseline samples to assess shifts in biomarkers. This limits the extent of comparisons that can be made between them. Finally, our work revealed some inter-animal variability in the inflammatory markers, microbial communities, and bacterial-derived metabolites that were assessed. This variability is to be expected among outbred animals and could further be due to factors, such as primate origin, genetics, and/or specific gut metabolism. The food provided was consistent between animals; thus, diet is unlikely to be a factor in the subtle inter-animal differences observed.

In conclusion, our study explored mucosal dysfunction and microbial dysbiosis as a potential mechanism underlying ZIKV pathogenesis in two species of macaques, as well as in ZIKV-infected humans. Overall, our study suggests that ZIKV infection could result in decreased functionality of the gut microbiome, which could, in turn, result in decreased gastrointestinal health, increased mucosal inflammation, and barrier damage. These disruptions could promote translocation of bacterial products into the periphery and contribute to the elevated inflammation observed in the periphery and CNS during ZIKV infection. Elevated inflammation may then induce upregulation of neopterin production and tryptophan catabolism in the CNS, which could contribute to the development of the neurological complications and deficits observed in patients infected with ZIKV. Additional studies are needed to better understand the mechanisms by which ZIKV and other viral infections, such as SARS-CoV-2 infection, may cause intestinal mucosal disruption—in particular, alterations in the production of microbial-derived metabolites—and whether these disruptions could promote mother-to-fetal transmission of ZIKV and the development of fetal neurological defects.

## MATERIALS AND METHODS

### Animals

Animals were housed in stainless steel cages with a 12/12 light cycle. All cage pans and animal rooms were cleaned daily and sanitized at least once every two weeks. Animals were provided with a commercial primate chow (Lab Diet, PMI Nutrition International) twice a day, with daily fruits and vegetables and water *ad libitum*. Male pigtail macaques were kept in run-through, paired housing throughout the entire study (M11121/Z11278; K11095/Z11157), while the females were in individual cages for the duration of the study. Environmental enrichment consisted of novel food items, foraging opportunities, and destructible and indestructible manipulanda. For minor procedures (Zika inoculation, blood, and rectal swab collection), animals were anesthetized with ketamine (10 mg/kg) and dexmedetomidine (0.017 mg/kg). Of note, on day seven of the study, female pigtail macaques had ZIKV-associated weight loss, which resulted in an overdraw of blood by 1–3 mL in these four animals relative to IACUC guidelines of 10 mL/kg/week. For more involved sample collection, including CSF collection, general anesthesia was maintained with isoflurane by inhalation, and post-operative analgesia was provided. Euthanasia was performed via an IV overdose (>75 mg/kg) of pentobarbital in accordance with the recommendations in the Guidelines for the Euthanasia of Animals set forth by the Panel on Euthanasia of the American Veterinary Medical Association (AVMA).

Existing plasma and CSF samples from rhesus macaques (*Macaca mulatta*; *n* = 8), collected in a previous study (4) conducted by Dr. D.H. Barouch at the Center for Virology and Vaccine Research at Beth Israel Deaconess Medical Center, were used in the present study. Briefly, rhesus macaques were housed and cared for at Bioqual, Rockville, MD, under a protocol approved by Bioqual and the IACUC of Beth Israel Deaconess Medical Center, Harvard Medical School. As previously described, all animals were housed in single cages throughout the study and had not been previously enrolled in any other prior studies.

### NHP sample collection and processing

As previously described (24), eight healthy pigtail macaques (cohort 1: four non-pregnant females, 8–10 years, 6–8 kg; cohort 2: 4 males, 4–6 years, 10–12 kg) were subcutaneously infected with 5 × 10^5^ plaque-forming units (PFU) of a Brazilian Fortaleza isolate of Zika virus (Brazil_2015_MG, GenBank: KX811222.1). Working stocks of ZIKV for the infection inoculum were grown and plaque-purified, as previously described (24). All animals were pre-screened for the presence of antibodies to West Nile, Dengue, and Zika viruses, and all were negative except a single female animal (L07226), who was seropositive for West Nile. Antibiotics were not administered throughout the entire study timeline and were not administered within a year of study enrollment, according to WaNPRC Animal History Reports. Diarrhea was not recorded in the Animal History Reports for any of the pigtail macaques during the time of this study. Study time points were referred to as the number of days post-ZIKV inoculation (dpi). Although male and female cohorts were sampled at different times, all sedation/sampling protocols remained the same between groups. Cerebrospinal fluid (CSF) was collected 10 days prior to inoculation and on days 7, 14, 21, and 28 dpi. To do this, animals were fasted for 24 hours prior to sedation, and CSF was collected and cryopreserved neat at −80℃ for later analysis. Non-invasive samples, including stool, rectal and vaginal swabs, and peripheral blood, were taken 10 and 4 days before ZIKV inoculation and at days 1–4, 7, 14, 21, and 28 dpi. Stool, rectal, and vaginal swabs were cryopreserved at −80℃ for later analysis. Blood samples were collected via venipuncture into EDTA vacutainers (BD, Franklin Lakes, NJ). Plasma was separated from whole blood by density gradient centrifugation and cryopreserved neat at −80℃ for later analysis. PTM was euthanized and necropsied at either 28 or 29 dpi.

As previously described (4), eight Indian-origin, healthy rhesus macaques (five females: 4–5 years, 4–5 kg; three males: 4–5 years, 5–6 kg) were subcutaneously infected with 1 × 10^3^ to 1 × 10^6^ PFU of ZIKV-BR 2015 and sampled at days 0 (pre-inoculation), 2, 7, 14, and 35 post-ZIKV. Animals were tested for baseline flavivirus-neutralizing antibodies. Antibiotic treatments were not administered to these animals, and of record, no GI symptoms (diarrhea) occurred throughout the study. CSF and plasma were collected at day 0 before inoculation (baseline time point), and at 2, 7, 14, and 35 dpi. CSF was cryopreserved neat; plasma was separated from EDTA whole blood by density gradient centrifugation.

### Human samples

Metadata for human serum samples included age, gender, place of exposure, date of illness onset, signs, and symptoms, as well as days post-onset (DPO) for each collection time point. Study subject ages ranged from 15 to 67 years old. Sex was majority female, with only 2/20 being males. Diagnostic tests were performed to verify ZIKV infection, such as qPCR and CDC lab diagnostics. Samples were grouped into either 1–14 DPO or post-infection (15–156 DPO). Five samples were grouped as 1–14 DPO, and the remaining 15 samples were in the post-infection group. To reconstitute the lyophilized human serum, samples were resuspended in the original volume indicated on the vial using Hyclone phosphate buffered saline (PBS; GE Healthcare Life Sciences; Pittsburgh, PA). The volume of samples ranged from 250 μL to 500 μL. Samples were vortexed until dissolved into solution. Finally, un-lyophilized plasma samples collected from healthy donors at the University of Washington/Fred Hutch Center for AIDS Research group at Harborview Medical Center were used as uninfected controls.

### Plasma assays

Soluble factors were assessed via enzyme-linked immunosorbent assay (ELISA) using the following commercially available kits: for soluble CD14 (sCD14), a Quantikine ELISA human sCD14 immunoassay from R&D Systems Inc. (Minneapolis, MN); for lipopolysaccharide binding protein (LBP), an ELISA kit from Biometic (Brixen, Italy); for monkey C-reactive protein (CRP), an ELISA kit from Life Diagnostics (West Chester, PA); and for zonulin, an ELISA kit from FineTest (Wuhan, China). Cytokines, chemokines, and growth factors were evaluated using xMAP Luminex with 23 targets (GCSF, GMCSF, IFN-γ, IL-1β, IL-1RA, IL-2, IL-4, IL-5, IL-6, IL-8, IL-10, IL12p40/IL23p40, IL-13, IL-15, IL-17A, IL-18, MCP-1, MIP-1α, MIP-1β, sCD40L, TGF-α, TNF-α, and VEGF) as previously described (24). Assays were completed according to the manufacturer’s recommended protocols, and all samples were assessed in duplicate. Samples were diluted for each assay as follows: for sCD14, plasma samples were diluted 1:200, and CSF samples were diluted 1:5; for LBP, plasma samples were diluted 1:2.67; and for CRP, plasma was diluted 1:1,000. Data were collected using an iMark microplate reader from Bio-Rad (Hercules, CA). Zika virus RNA copies/mL were measured in plasma via RT-qPCR.

### 16S rRNA sequencing

#### Genomic DNA extraction and sequencing

Genomic DNA was extracted from rectal swab specimens using lysis buffer, chicken egg white lysozyme, SDS, and an RNA/DNA AllPrep Kit: DNeasy Blood and Tissue Kit (Qiagen, Valencia, CA). DNA for 16S rRNA sequencing was processed using the Earth Microbiome Project protocols (28, 41–44) with the following modifications. During the library preparation, each DNA sample was amplified in triplicate using the FailSafe PCR System (Epicenter, WI) and the 515FB-806RB primer pair to generate a 400 bp amplicon from the V4 variable regions of the 16S rRNA gene. The triplicate reactions were pooled, quantified using Qubit dsDNA High Sensitivity Assay Kit (ThermoFisher Scientific, Waltham, MA), and visualized using a LabChip GX (PerkinElmer, MA). Ten nanograms of each library were pooled. The pooled library was cleaned using MO BIO UltraClean PCR Clean-Up Kit (MO BIO, Carlsbad, CA) and quantified using the KAPA Library Quantification Kit (KAPA Biosystems, Wilmington, MA). Sequencing was carried out as detailed in the EMP protocol: specifically, 7 pM of the pooled library with 30% PhiX phage as a control was sequenced using a 300-cycle Illumina MiSeq Kit (Illumina, Inc., San Diego, CA), generating 150 bp paired-end reads.

#### Analysis of gene sequence data

Raw 16S rRNA gene sequence data were processed using the QIIME 2 software package (version 2018.2.0) via the DADA2 (79) pipeline option which performs quality filtering and identification of exact amplicon sequence variants (ASVs). We then used the Greengenes database (release gg-13-8-99-515-806-nb-classifier) for taxonomic assignments. ASVs that were unassigned at the Kingdom level were filtered out, and we removed ASVs that were present in only one sample. We then performed principal coordinate analysis (PCoA) on log-normalized counts and generated relative abundance plots as a percentage of counts of the top 20 genera in each tissue type using the phyloseq R package 1.22 (80). To identify differences in taxa abundance relative to baseline, we used the DESeq2 R package 1.18 (81) to fit taxa abundances into a negative binomial generalized linear model (GLM), with parametric fitting of dispersions to the mean and *P*-values calculated using Wald significance tests. Significant differences were defined as ASVs with thresholds of adjusted *P*-value < 0.05 and a greater than 1.5 log_2_-fold-change difference from baseline in at least one time point.

### Mass spectrometry

#### Liquid chromatography tandem mass spectrometry (LC-MS/MS)

Tryptophan (L-Trp), kynurenine (L-Kyn), serotonin, and neopterin (D-(+)-neopterin) concentrations were determined in plasma and CSF by LC-MS/MS. This method was validated for its sensitivity, selectivity, accuracy, precision, matrix effects, recovery, and stability. Ten microliters of sample was spiked with 10 μL of internal standard (IS; L-Trp-d5) and 10 μL acidified mobile phase (AMP; 0.2%FA/0.05%TFA/1% acetonitrile [ACN; Millipore-Sigma] in water). Subsequently, 150 μL of ice-cold methanol (MeOH; Millipore Sigma) was added, and this mixture was allowed to rest for 30 min at −20°C to support protein precipitation. After centrifugation (3000 × *g*, RT, 10 min), supernatants were removed and evaporated to dryness under a gentle stream of nitrogen. Dry extracts were reconstituted in 40 μL AMP. Twenty microliters of sample was injected into the LC-MS/MS. Chromatographic separation was achieved using a gradient elution with a Chromolith Performance RP-C18 column (Millipore-Sigma). The column was maintained at 25°C throughout. Samples were subjected to positive electrospray ionization (ESI) and detected via multiple reaction monitoring (MRM) using a LC-MS/MS system (Agilent Technologies 6460 QQQ/MassHunter; Palo Alto, CA). Each transition was monitored with a 150 ms dwell time. Mobile phase A is 0.1% formic acid in H_2_O, and mobile phase B is 0.1% formic acid in methanol. During pre-study validation, calibration curves were defined in multiple runs on the basis of triplicate assays of spiked samples and quality control (QC) samples. Calibration standards were prepared and ranged from 0.05 to 1,000 nmol/L with an inter- and intra-day precision and accuracy of ≤10.1% with an *r*^2^ value of 0.9973 ± 0.0037. The LOD for all analytes was 0.05 nmol/L with an LOQ of 0.1 nmol/L. Quantification was performed using MRM of the transitions of m/z 205.1→118.0, 209.1→94.1, 254.1→206.1, 177.1→160.0, 210.1→122.1 for tryptophan, kynurenine, neopterin, serotonin, and the internal standard (IS) tryptophan-d5, respectively.

#### Gas chromatography mass spectrometry (GC-MS)

Concentrations of six short-chain fatty acids (SCFAs), including acetate (acetic acid), propionate (propionic acid), isobutyrate (isobutyric acid), butyrate (butyric acid), isovalerate (isovaleric acid), and valerate (valeric acid), were determined in stool by GC-MS. This method was validated for its sensitivity, selectivity, accuracy, precision, matrix effects, recovery, and stability. Total short-chain fatty acids included all six acids. A 1 mL of acidified water (pH ~2.5, 9% formic acid; Millipore-Sigma) was added per 100 mg of stool sample. Samples were vortexed and centrifuged at max speed for 5 min. Three hundred microliters of supernatant was removed and transferred to a fresh, sterile, microcentrifuge tube. Thirty microliters of internal standard (IS; 2-methyl-valeric acid) was added to each sample. Three hundred microliters of ethyl acetate was added to each sample, vortexed for 10 min, and centrifuged at max speed for 5 min. Two hundred microliters was carefully removed from the upper organic phase and transferred into a glass insert and capped immediately with a non-pre-slit cap. Samples were stored at −20°C until they were collected on the GC-MS in batches. The GC-MS system consisted of an Agilent 5973N MSD EI/CI with 6890 GC and autosampler (Agilent Technologies). Acquisition was done using Chemstation software (Hewlett-Packard, Palo Alto, CA, USA). The GC was fitted with a polyethylene glycol (PEG) fused silica capillary column, Stabilwax/Carbowax (30 m, 0.32 mm id, 0.25 um film thickness), and helium was used as the carrier gas at 1 mL/min. An injection volume of 1 μL with an injection temperature of 250°C was used. For every three fecal samples injected, a blank sample with diethyl ether was inserted to minimize carryover effects. The column temperature was initially 100°C and held for 2 min, then increased to 200°C at 25°C/min, and kept at this temperature for 2 min (8 min total time). The detector was operated in electron impact ionization mode at an electron energy of 70 V, scanning 30–250 *m/z* range. The temperature of the ion source, quadrupole, and interface was 220°C, 140°C, and 270°C, respectively. During pre-study validation, calibration curves were defined in multiple runs on the basis of triplicate assays of spiked samples and QC samples. Calibration standards were prepared and ranged from 0.01 to 100 umol/L with an inter- and intra-day precision and accuracy of <7.4% with an *r*^2^ value of 0.998 ± 0.0013. The LOD for all SCFA was 0.05 uM. Quantification was performed using quantification of target ions of 60, 74, 88, 73, 87, and 73 for acetic, propionic, isobutyric, butyric, isovaleric, and valeric acid, respectively.

### Data and statistical analysis

Statistical analyses were performed using GraphPad Prism statistical software (version 7; GraphPad Software, San Diego, CA). For NHP studies, results obtained at the pre-infection time points (day −10 or day −5 depending on sample type) were compared to those obtained 7, 14, 21, and 27–29 dpi. The significance between pre-infection and multiple post-infection time points in PTM and RM was evaluated using a repeated measures ANOVA with the Geisser-Greenhouse correction and a Dunnett’s multiple comparisons post-test, where *P-*values of < 0.05 were considered significant. Repeated measures correlations between biomarkers and viral RNA copies/mL were conducted with the *rmcorr* package in R, where a false discovery rate (FDR) adjusted *q*-value was considered significant when *q* < 0.05 (R: version 4.4.0, *rmcorr*: version 0.7.0) (82). Biomarkers that were undetected in the samples and consequently set to the limit of detection, including IFN-γ, IL-1β, and zonulin, were removed prior to calculating correlations. Statistical analysis of human samples was performed by comparing the uninfected control group to either the ZIKV-infected group (1–14 DPO and post-infection). Statistical significance was evaluated using an ANOVA with a Dunnett’s multiple comparisons post-test, where *P-*values < 0.05 were considered significant. To assess correlations between SCFA levels and inflammatory markers, Spearman’s rank-order correlations were performed, and a Bonferroni correction was applied for multiple comparisons (*P* < 0.0006).

## Data Availability

Raw 16S sequencing reads were submitted to NCBI’s Sequence Read Archive under accession number PRJNA480675. Code and other data are available upon request.

## SUPPLEMENTAL MATERIAL

Supplemental figures and table jvi.01003-25-s0001.pdf Fig. S1 to S4 and Table S1.

ASM does not own the copyrights to Supplemental Material that may be linked to, or accessed through, an article. The authors have granted ASM a non-exclusive, world-wide license to publish the Supplemental Material files. Please contact the corresponding author directly for reuse.

## References

[1] França GVA, Schuler-Faccini L, Oliveira WK, Henriques CMP, Carmo EH, Pedi VD, Nunes ML, Castro MC, Serruya S, Silveira MF, Barros FC, Victora CG. 2016. Congenital Zika virus syndrome in Brazil: a case series of the first 1501 livebirths with complete investigation. Lancet 388:891–897. doi:10.1016/S0140-6736(16)30902-3 27372398

[2] da Silva IRF, Frontera JA, Bispo de Filippis AM, Nascimento OJM do, RIO-GBS-ZIKV Research Group. 2017. Neurologic complications associated with the Zika virus in Brazilian adults. JAMA Neurol 74:1190–1198. doi:10.1001/jamaneurol.2017.1703 28806453 PMC5710239

[3] Higgs S. 2016. Zika virus: emergence and emergency. Vector Borne Zoonotic Dis 16:75–76. doi:10.1089/vbz.2016.29001.hig 26824625

[4] Aid M, Abbink P, Larocca RA, Boyd M, Nityanandam R, Nanayakkara O, Martinot AJ, Moseley ET, Blass E, Borducchi EN, Chandrashekar A, Brinkman AL, Molloy K, Jetton D, Tartaglia LJ, Liu J, Best K, Perelson AS, De La Barrera RA, Lewis MG, Barouch DH. 2017. Zika virus persistence in the central nervous system and lymph nodes of rhesus monkeys. Cell 169:610–620. doi:10.1016/j.cell.2017.04.008 28457610 PMC5426912

[5] Brito Ferreira ML, Antunes de Brito CA, Moreira ÁJP, de Morais Machado MÍ, Henriques-Souza A, Cordeiro MT, de Azevedo Marques ET, Pena LJ. 2017. Guillain–Barré syndrome, acute disseminated encephalomyelitis and encephalitis associated with Zika virus infection in Brazil: detection of viral RNA and isolation of virus during late infection. Am J Trop Med Hyg 97:1405–1409. doi:10.4269/ajtmh.17-0106 29140242 PMC5817749

[6] Carteaux G, Maquart M, Bedet A, Contou D, Brugières P, Fourati S, Cleret de Langavant L, de Broucker T, Brun-Buisson C, Leparc-Goffart I, Mekontso Dessap A. 2016. Zika virus associated with meningoencephalitis. N Engl J Med 374:1595–1596. doi:10.1056/NEJMc1602964 26958738

[7] Muñoz LS, Barreras P, Pardo CA. 2016. Zika virus-associated neurological disease in the adult: Guillain-Barré syndrome, encephalitis, and myelitis. Semin Reprod Med 34:273–279. doi:10.1055/s-0036-1592066 27612158

[8] Rozé B, Najioullah F, Signate A, Apetse K, Brouste Y, Gourgoudou S, Fagour L, Abel S, Hochedez P, Cesaire R, Cabié A, Neuro-Zika Working Group of Martinique. 2016. Zika virus detection in cerebrospinal fluid from two patients with encephalopathy, Martinique, February 2016. Euro Surveill 21:30205. doi:10.2807/1560-7917.ES.2016.21.16.30205 27123558

[9] Figueiredo CP, Barros-Aragão FGQ, Neris RLS, Frost PS, Soares C, Souza INO, Zeidler JD, Zamberlan DC, de Sousa VL, Souza AS, Guimarães ALA, Bellio M, Marcondes de Souza J, Alves-Leon SV, Neves GA, Paula-Neto HA, Castro NG, De Felice FG, Assunção-Miranda I, Clarke JR, Da Poian AT, Ferreira ST. 2019. Zika virus replicates in adult human brain tissue and impairs synapses and memory in mice. Nat Commun 10:3890. doi:10.1038/s41467-019-11866-7 31488835 PMC6728367

[10] Mécharles S, Herrmann C, Poullain P, Tran T-H, Deschamps N, Mathon G, Landais A, Breurec S, Lannuzel A. 2016. Acute myelitis due to Zika virus infection. Lancet 387:1481. doi:10.1016/S0140-6736(16)00644-9 26946926

[11] Soares CN, Brasil P, Carrera RM, Sequeira P, de Filippis AB, Borges VA, Theophilo F, Ellul MA, Solomon T. 2016. Fatal encephalitis associated with Zika virus infection in an adult. J Clin Virol 83:63–65. doi:10.1016/j.jcv.2016.08.297 27598870

[12] Kelley TW, Prayson RA, Ruiz AI, Isada CM, Gordon SM. 2003. The neuropathology of West Nile virus meningoencephalitis. A report of two cases and review of the literature. Am J Clin Pathol 119:749–753. doi:10.1309/PU4R-76JJ-MG1F-81RP 12760295

[13] Johnson RT, Burke DS, Elwell M, Leake CJ, Nisalak A, Hoke CH, Lorsomrudee W. 1985. Japanese encephalitis: immunocytochemical studies of viral antigen and inflammatory cells in fatal cases. Ann Neurol 18:567–573. doi:10.1002/ana.410180510 3000282

[14] Eisenhut M. 2013. Neopterin in diagnosis and monitoring of infectious diseases. J Biomark 2013:196432. doi:10.1155/2013/196432 26317013 PMC4437389

[15] Hamerlinck FF. 1999. Neopterin: a review. Exp Dermatol 8:167–176. doi:10.1111/j.1600-0625.1999.tb00367.x 10389633

[16] Hagberg L, Dotevall L, Norkrans G, Larsson M, Wachter H, Fuchs D. 1993. Cerebrospinal fluid neopterin concentrations in central nervous system infection. J Infect Dis 168:1285–1288. doi:10.1093/infdis/168.5.1285 8228365

[17] Jones SP, Franco NF, Varney B, Sundaram G, Brown DA, de Bie J, Lim CK, Guillemin GJ, Brew BJ. 2015. Expression of the kynurenine pathway in human peripheral blood mononuclear cells: implications for inflammatory and neurodegenerative disease. PLoS ONE 10:e0131389. doi:10.1371/journal.pone.0131389 26114426 PMC4482723

[18] Mehraj V, Routy J-P. 2015. Tryptophan catabolism in chronic viral infections: handling uninvited guests. Int J Tryptophan Res 8:41–48. doi:10.4137/IJTR.S26862 26309411 PMC4527356

[19] Orhan F, Bhat M, Sandberg K, Ståhl S, Piehl F, Svensson C, Erhardt S, Schwieler L, Karolinska Schizophrenia Project (KaSP) consortium. 2016. Tryptophan metabolism along the kynurenine pathway downstream of Toll-like receptor stimulation in peripheral monocytes. Scand J Immunol 84:262–271. doi:10.1111/sji.12479 27607184

[20] Werner ER, Bitterlich G, Fuchs D, Hausen A, Reibnegger G, Szabo G, Dierich MP, Wachter H. 1987. Human macrophages degrade tryptophan upon induction by interferon-gamma. Life Sci 41:273–280. doi:10.1016/0024-3205(87)90149-4 3110526

[21] Gostner JM, Geisler S, Stonig M, Mair L, Sperner-Unterweger B, Fuchs D. 2020. Tryptophan metabolism and related pathways in psychoneuroimmunology: the impact of nutrition and lifestyle. Neuropsychobiology 79:89–99. doi:10.1159/000496293 30808841

[22] Stone TW, Mackay GM, Forrest CM, Clark CJ, Darlington LG. 2003. Tryptophan metabolites and brain disorders. Clin Chem Lab Med 41:852–859. doi:10.1515/CCLM.2003.129 12940508

[23] Widner B, Laich A, Sperner-Unterweger B, Ledochowski M, Fuchs D. 2002. Neopterin production, tryptophan degradation, and mental depression—What is the link? Brain Behav Immun 16:590–595. doi:10.1016/S0889-1591(02)00006-5 12401473

[24] O’Connor MA, Tisoncik-Go J, Lewis TB, Miller CJ, Bratt D, Moats CR, Edlefsen PT, Smedley J, Klatt NR, Gale M Jr, Fuller DH. 2018. Early cellular innate immune responses drive Zika viral persistence and tissue tropism in pigtail macaques. Nat Commun 9:3371. doi:10.1038/s41467-018-05826-w 30135445 PMC6105614

[25] Yockey LJ, Varela L, Rakib T, Khoury-Hanold W, Fink SL, Stutz B, Szigeti-Buck K, Van den Pol A, Lindenbach BD, Horvath TL, Iwasaki A. 2016. Vaginal exposure to Zika virus during pregnancy leads to fetal brain infection. Cell 166:1247–1256. doi:10.1016/j.cell.2016.08.004 27565347 PMC5006689

[26] Dillon SM, Lee EJ, Kotter CV, Austin GL, Dong Z, Hecht DK, Gianella S, Siewe B, Smith DM, Landay AL, Robertson CE, Frank DN, Wilson CC. 2014. An altered intestinal mucosal microbiome in HIV-1 infection is associated with mucosal and systemic immune activation and endotoxemia. Mucosal Immunol 7:983–994. doi:10.1038/mi.2013.116 24399150 PMC4062575

[27] Vujkovic-Cvijin I, Dunham RM, Iwai S, Maher MC, Albright RG, Broadhurst MJ, Hernandez RD, Lederman MM, Huang Y, Somsouk M, Deeks SG, Hunt PW, Lynch SV, McCune JM. 2013. Dysbiosis of the gut microbiota is associated with HIV disease progression and tryptophan catabolism. Sci Transl Med 5:193ra91. doi:10.1126/scitranslmed.3006438 PMC4094294 23843452

[28] Zevin AS, McKinnon L, Burgener A, Klatt NR. 2016. Microbial translocation and microbiome dysbiosis in HIV-associated immune activation. Curr Opin HIV AIDS 11:182–190. doi:10.1097/COH.0000000000000234 26679414 PMC4752849

[29] Inoue T, Nakayama J, Moriya K, Kawaratani H, Momoda R, Ito K, Iio E, Nojiri S, Fujiwara K, Yoneda M, Yoshiji H, Tanaka Y. 2018. Gut dysbiosis associated with hepatitis C virus infection. Clin Infect Dis 67:869–877. doi:10.1093/cid/ciy205 29718124

[30] Belkaid Y, Hand TW. 2014. Role of the microbiota in immunity and inflammation. Cell 157:121–141. doi:10.1016/j.cell.2014.03.011 24679531 PMC4056765

[31] Gardner MB, Luciw PA. 2008. Macaque models of human infectious disease. ILAR J 49:220–255. doi:10.1093/ilar.49.2.220 18323583 PMC7108592

[32] Hirsch AJ, Smith JL, Haese NN, Broeckel RM, Parkins CJ, Kreklywich C, DeFilippis VR, Denton M, Smith PP, Messer WB, Colgin LMA, Ducore RM, Grigsby PL, Hennebold JD, Swanson T, Legasse AW, Axthelm MK, MacAllister R, Wiley CA, Nelson JA, Streblow DN. 2017. Zika virus infection of rhesus macaques leads to viral persistence in multiple tissues. PLOS Pathog 13:e1006219. doi:10.1371/journal.ppat.1006219 28278237 PMC5344528

[33] Dudley DM, Aliota MT, Mohr EL, Weiler AM, Lehrer-Brey G, Weisgrau KL, Mohns MS, Breitbach ME, Rasheed MN, Newman CM, et al.. 2016. A rhesus macaque model of Asian-lineage Zika virus infection. Nat Commun 7:12204. doi:10.1038/ncomms12204 27352279 PMC4931337

[34] Styer LM, Kent KA, Albright RG, Bennett CJ, Kramer LD, Bernard KA. 2007. Mosquitoes inoculate high doses of West Nile virus as they probe and feed on live hosts. PLoS Pathog 3:1262–1270. doi:10.1371/journal.ppat.0030132 17941708 PMC1976553

[35] Hagberg L, Cinque P, Gisslen M, Brew BJ, Spudich S, Bestetti A, Price RW, Fuchs D. 2010. Cerebrospinal fluid neopterin: an informative biomarker of central nervous system immune activation in HIV-1 infection. AIDS Res Ther 7:15. doi:10.1186/1742-6405-7-15 20525234 PMC2890504

[36] Viaccoz A, Ducray F, Tholance Y, Barcelos GK, Thomas-Maisonneuve L, Ghesquières H, Meyronet D, Quadrio I, Cartalat-Carel S, Louis-Tisserand G, Jouanneau E, Guyotat J, Honnorat J, Perret-Liaudet A. 2015. CSF neopterin level as a diagnostic marker in primary central nervous system lymphoma. Neuro Oncol 17:1497–1503. doi:10.1093/neuonc/nov092 26014047 PMC4648303

[37] Pepys MB, Hirschfield GM. 2003. C-reactive protein: a critical update. J Clin Invest 111:1805–1812. doi:10.1172/JCI18921 12813013 PMC161431

[38] Zhou Y-H, Sun L, Chen J, Sun W-W, Ma L, Han Y, Jin X, Zhao Q-X, Li T, Lu H, Qiu X, Wang J-H. 2019. Tryptophan metabolism activates aryl hydrocarbon receptor-mediated pathway to promote HIV-1 infection and reactivation. mBio 10:e02591-19. doi:10.1128/mBio.02591-19 31848275 PMC6918076

[39] Stone TW, Darlington LG. 2013. The kynurenine pathway as a therapeutic target in cognitive and neurodegenerative disorders. Br J Pharmacol 169:1211–1227. doi:10.1111/bph.12230 23647169 PMC3831703

[40] Jenkins TA, Nguyen JCD, Polglaze KE, Bertrand PP. 2016. Influence of tryptophan and serotonin on mood and cognition with a possible role of the gut-brain axis. Nutrients 8:56. doi:10.3390/nu8010056 26805875 PMC4728667

[41] Moon MS, Quinn G, Townsend EC, Ali RO, Zhang GY, Bradshaw A, Hill K, Guan H, Hamilton D, Kleiner DE, Koh C, Heller T. 2019. Bacterial translocation and host immune activation in chronic hepatitis C infection. Open Forum Infect Dis 6:fz255. doi:10.1093/ofid/ofz255 PMC6667717 31363763

[42] van de Weg CAM, Pannuti CS, de Araújo ESA, van den Ham H-J, Andeweg AC, Boas LSV, Felix AC, Carvalho KI, de Matos AM, Levi JE, Romano CM, Centrone CC, de Lima Rodrigues CL, Luna E, van Gorp ECM, Osterhaus ADME, Martina BEE, Kallas EG. 2013. Microbial translocation is associated with extensive immune activation in dengue virus infected patients with severe disease. PLoS Negl Trop Dis 7:e2236. doi:10.1371/journal.pntd.0002236 23717702 PMC3662706

[43] Sandler N.G., Douek DC. 2012. Microbial translocation in HIV infection: causes, consequences and treatment opportunities. Nat Rev Microbiol 10:655–666. doi:10.1038/nrmicro2848 22886237

[44] Sandler NG, Wand H, Roque A, Law M, Nason MC, Nixon DE, Pedersen C, Ruxrungtham K, Lewin SR, Emery S, Neaton JD, Brenchley JM, Deeks SG, Sereti I, Douek DC, INSIGHT SMART Study Group. 2011. Plasma levels of soluble CD14 independently predict mortality in HIV infection. J Infect Dis 203:780–790. doi:10.1093/infdis/jiq118 21252259 PMC3071127

[45] Klatt NR, Funderburg NT, Brenchley JM. 2013. Microbial translocation, immune activation, and HIV disease. Trends Microbiol 21:6–13. doi:10.1016/j.tim.2012.09.001 23062765 PMC3534808

[46] Wright SD, Ramos RA, Tobias PS, Ulevitch RJ, Mathison JC. 1990. CD14, a receptor for complexes of lipopolysaccharide (LPS) and LPS binding protein. Science 249:1431–1433. doi:10.1126/science.1698311 1698311

[47] Bufler P, Stiegler G, Schuchmann M, Hess S, Krüger C, Stelter F, Eckerskorn C, Schütt C, Engelmann H. 1995. Soluble lipopolysaccharide receptor (CD14) is released via two different mechanisms from human monocytes and CD14 transfectants. Eur J Immunol 25:604–610. doi:10.1002/eji.1830250244 7533093

[48] Shive CL, Jiang W, Anthony DD, Lederman MM. 2015. Soluble CD14 is a nonspecific marker of monocyte activation. AIDS 29:1263–1265. doi:10.1097/QAD.0000000000000735 26035325 PMC4452959

[49] Jespersen S, Pedersen KK, Anesten B, Zetterberg H, Fuchs D, Gisslén M, Hagberg L, Trøseid M, Nielsen SD. 2016. Soluble CD14 in cerebrospinal fluid is associated with markers of inflammation and axonal damage in untreated HIV-infected patients: a retrospective cross-sectional study. BMC Infect Dis 16:176. doi:10.1186/s12879-016-1510-6 27103116 PMC4839160

[50] Schumann RR, Zweigner J. 1999. A novel acute-phase marker: lipopolysaccharide binding protein (LBP). Clin Chem Lab Med 37:271–274. doi:10.1515/CCLM.1999.047 10353471

[51] Tsukamoto H, Takeuchi S, Kubota K, Kobayashi Y, Kozakai S, Ukai I, Shichiku A, Okubo M, Numasaki M, Kanemitsu Y, Matsumoto Y, Nochi T, Watanabe K, Aso H, Tomioka Y. 2018. Lipopolysaccharide (LPS)-binding protein stimulates CD14-dependent Toll-like receptor 4 internalization and LPS-induced TBK1-IKKϵ-IRF3 axis activation. J Biol Chem 293:10186–10201. doi:10.1074/jbc.M117.796631 29760187 PMC6028956

[52] Tobias PS, Ulevitch RJ. 1993. Lipopolysaccharide binding protein and CD14 in LPS dependent macrophage activation. Immunobiology 187:227–232. doi:10.1016/S0171-2985(11)80341-4 7687234

[53] Pelsers MMAL, Namiot Z, Kisielewski W, Namiot A, Januszkiewicz M, Hermens WT, Glatz JFC. 2003. Intestinal-type and liver-type fatty acid-binding protein in the intestine. Tissue distribution and clinical utility. Clin Biochem 36:529–535. doi:10.1016/s0009-9120(03)00096-1 14563446

[54] Saffouri GB, Shields-Cutler RR, Chen J, Yang Y, Lekatz HR, Hale VL, Cho JM, Battaglioli EJ, Bhattarai Y, Thompson KJ, Kalari KK, Behera G, Berry JC, Peters SA, Patel R, Schuetz AN, Faith JJ, Camilleri M, Sonnenburg JL, Farrugia G, Swann JR, Grover M, Knights D, Kashyap PC. 2019. Small intestinal microbial dysbiosis underlies symptoms associated with functional gastrointestinal disorders. Nat Commun 10:2012. doi:10.1038/s41467-019-09964-7 31043597 PMC6494866

[55] Trøseid M, Andersen GØ, Broch K, Hov JR. 2020. The gut microbiome in coronary artery disease and heart failure: current knowledge and future directions. EBioMedicine 52:102649. doi:10.1016/j.ebiom.2020.102649 32062353 PMC7016372

[56] Sochocka M, Donskow-Łysoniewska K, Diniz BS, Kurpas D, Brzozowska E, Leszek J. 2019. The gut microbiome alterations and inflammation-driven pathogenesis of Alzheimer’s disease-a critical review. Mol Neurobiol 56:1841–1851. doi:10.1007/s12035-018-1188-4 29936690 PMC6394610

[57] Toor D, Wsson MK, Kumar P, Karthikeyan G, Kaushik NK, Goel C, Singh S, Kumar A, Prakash H. 2019. Dysbiosis disrupts gut immune homeostasis and promotes gastric diseases. Int J Mol Sci 20:2432. doi:10.3390/ijms20102432 31100929 PMC6567003

[58] Zeng MY, Inohara N, Nuñez G. 2017. Mechanisms of inflammation-driven bacterial dysbiosis in the gut. Mucosal Immunol 10:18–26. doi:10.1038/mi.2016.75 27554295 PMC5788567

[59] White JP, Xiong S, Malvin NP, Khoury-Hanold W, Heuckeroth RO, Stappenbeck TS, Diamond MS. 2018. Intestinal dysmotility syndromes following systemic infection by flaviviruses. Cell 175:1198–1212. doi:10.1016/j.cell.2018.08.069 30293866 PMC6309989

[60] den Besten G, van Eunen K, Groen AK, Venema K, Reijngoud D-J, Bakker BM. 2013. The role of short-chain fatty acids in the interplay between diet, gut microbiota, and host energy metabolism. J Lipid Res 54:2325–2340. doi:10.1194/jlr.R036012 23821742 PMC3735932

[61] Li M, van Esch BCAM, Wagenaar GTM, Garssen J, Folkerts G, Henricks PAJ. 2018. Pro- and anti-inflammatory effects of short chain fatty acids on immune and endothelial cells. Eur J Pharmacol 831:52–59. doi:10.1016/j.ejphar.2018.05.003 29750914

[62] Morrison DJ, Preston T. 2016. Formation of short chain fatty acids by the gut microbiota and their impact on human metabolism. Gut Microbes 7:189–200. doi:10.1080/19490976.2015.1134082 26963409 PMC4939913

[63] Fukumoto S, Tatewaki M, Yamada T, Fujimiya M, Mantyh C, Voss M, Eubanks S, Harris M, Pappas TN, Takahashi T. 2003. Short-chain fatty acids stimulate colonic transit via intraluminal 5-HT release in rats. Am J Physiol Regul Integr Comp Physiol 284:R1269–76. doi:10.1152/ajpregu.00442.2002 12676748

[64] Lamas B, Richard ML, Leducq V, Pham H-P, Michel M-L, Da Costa G, Bridonneau C, Jegou S, Hoffmann TW, Natividad JM, Brot L, Taleb S, Couturier-Maillard A, Nion-Larmurier I, Merabtene F, Seksik P, Bourrier A, Cosnes J, Ryffel B, Beaugerie L, Launay J-M, Langella P, Xavier RJ, Sokol H. 2016. CARD9 impacts colitis by altering gut microbiota metabolism of tryptophan into aryl hydrocarbon receptor ligands. Nat Med 22:598–605. doi:10.1038/nm.4102 27158904 PMC5087285

[65] Samarasekera U, Triunfol M. 2016. Concern over Zika virus grips the world. Lancet 387:521–524. doi:10.1016/S0140-6736(16)00257-9 26852261

[66] Yang Y, Jobin C. 2014. Microbial imbalance and intestinal pathologies: connections and contributions. Dis Model Mech 7:1131–1142. doi:10.1242/dmm.016428 25256712 PMC4174524

[67] Morris G, Berk M, Carvalho A, Caso JR, Sanz Y, Walder K, Maes M. 2017. The role of the microbial metabolites including tryptophan catabolites and short chain fatty acids in the pathophysiology of immune-inflammatory and neuroimmune disease. Mol Neurobiol 54:4432–4451. doi:10.1007/s12035-016-0004-2 27349436

[68] van de Poll MCG, Derikx JPM, Buurman WA, Peters WHM, Roelofs HMJ, Wigmore SJ, Dejong CH. 2007. Liver manipulation causes hepatocyte injury and precedes systemic inflammation in patients undergoing liver resection. World J Surg 31:2033–2038. doi:10.1007/s00268-007-9182-4 17668263 PMC2039834

[69] Burdo TH, Lackner A, Williams KC. 2013. Monocyte/macrophages and their role in HIV neuropathogenesis. Immunol Rev 254:102–113. doi:10.1111/imr.12068 23772617 PMC3704190

[70] Erkenekli K, Keskin U, Uysal B, Kurt YG, Sadir S, Çayci T, Ergün A, Erkaya S, Danişman N, Uygur D. 2015. Levels of neopterin and C-reactive protein in pregnant women with fetal growth restriction. J Obstet Gynaecol 35:225–228. doi:10.3109/01443615.2014.948818 25140392

[71] Guillemin GJ, Kerr SJ, Brew BJ. 2005. Involvement of quinolinic acid in AIDS dementia complex. Neurotox Res 7:103–123. doi:10.1007/BF03033781 15639803

[72] Goeden N, Velasquez J, Arnold KA, Chan Y, Lund BT, Anderson GM, Bonnin A. 2016. Maternal inflammation disrupts fetal neurodevelopment via increased placental output of serotonin to the fetal brain. J Neurosci 36:6041–6049. doi:10.1523/JNEUROSCI.2534-15.2016 27251625 PMC4887568

[73] Richard DM, Dawes MA, Mathias CW, Acheson A, Hill-Kapturczak N, Dougherty DM. 2009. L-tryptophan: basic metabolic functions, behavioral research and therapeutic indications. Int J Tryptophan Res 2:45–60. doi:10.4137/ijtr.s2129 20651948 PMC2908021

[74] Murugesan S, Nirmalkar K, Hoyo-Vadillo C, García-Espitia M, Ramírez-Sánchez D, García-Mena J. 2018. Gut microbiome production of short-chain fatty acids and obesity in children. Eur J Clin Microbiol Infect Dis 37:621–625. doi:10.1007/s10096-017-3143-0 29196878

[75] Soldavini J, Kaunitz JD. 2013. Pathobiology and potential therapeutic value of intestinal short-chain fatty acids in gut inflammation and obesity. Dig Dis Sci 58:2756–2766. doi:10.1007/s10620-013-2744-4 23839339 PMC4317286

[76] Dillon SM, Kibbie J, Lee EJ, Guo K, Santiago ML, Austin GL, Gianella S, Landay AL, Donovan AM, Frank DN, McCarter MD, Wilson CC. 2017. Low abundance of colonic butyrate-producing bacteria in HIV infection is associated with microbial translocation and immune activation. AIDS 31:511–521. doi:10.1097/QAD.0000000000001366 28002063 PMC5263163

[77] Parada Venegas D, De la Fuente MK, Landskron G, González MJ, Quera R, Dijkstra G, Harmsen HJM, Faber KN, Hermoso MA. 2019. Short chain fatty acids (SCFAs)-mediated gut epithelial and immune regulation and its relevance for inflammatory bowel diseases. Front Immunol 10:277. doi:10.3389/fimmu.2019.00277 30915065 PMC6421268

[78] Qing Y, Xie H, Su C, Wang Y, Yu Q, Pang Q, Cui F. 2019. Gut microbiome, short-chain fatty acids, and mucosa injury in young adults with human immunodeficiency virus infection. Dig Dis Sci 64:1830–1843. doi:10.1007/s10620-018-5428-2 30560340

[79] Callahan BJ, McMurdie PJ, Rosen MJ, Han AW, Johnson AJA, Holmes SP. 2016. DADA2: high-resolution sample inference from Illumina amplicon data. Nat Methods 13:581–583. doi:10.1038/nmeth.3869 27214047 PMC4927377

[80] McMurdie PJ, Holmes S. 2013. phyloseq: an R package for reproducible interactive analysis and graphics of microbiome census data. PLoS ONE 8:e61217. doi:10.1371/journal.pone.0061217 23630581 PMC3632530

[81] Love MI, Huber W, Anders S. 2014. Moderated estimation of fold change and dispersion for RNA-seq data with DESeq2. Genome Biol 15:550. doi:10.1186/s13059-014-0550-8 25516281 PMC4302049

[82] Bakdash JZ, Marusich LR. 2017. Repeated measures correlation. Front Psychol 8:456. doi:10.3389/fpsyg.2017.00456 28439244 PMC5383908

