# Supplementary material for: Elevated peripheral and nervous system inflammation is associated with decreased short-chain fatty acid levels in Zika virus-infected macaques
Source: J Virol. 2025 Aug 19;99(9):e01003-25. doi: 10.1128/jvi.01003-25 (PMC12455978; doi:10.1128/jvi.01003-25)
Supplement: Supplemental figures and table — Fig. S1 to S4 and Table S1. [file jvi.01003-25-s0001.pdf]

Figure S1

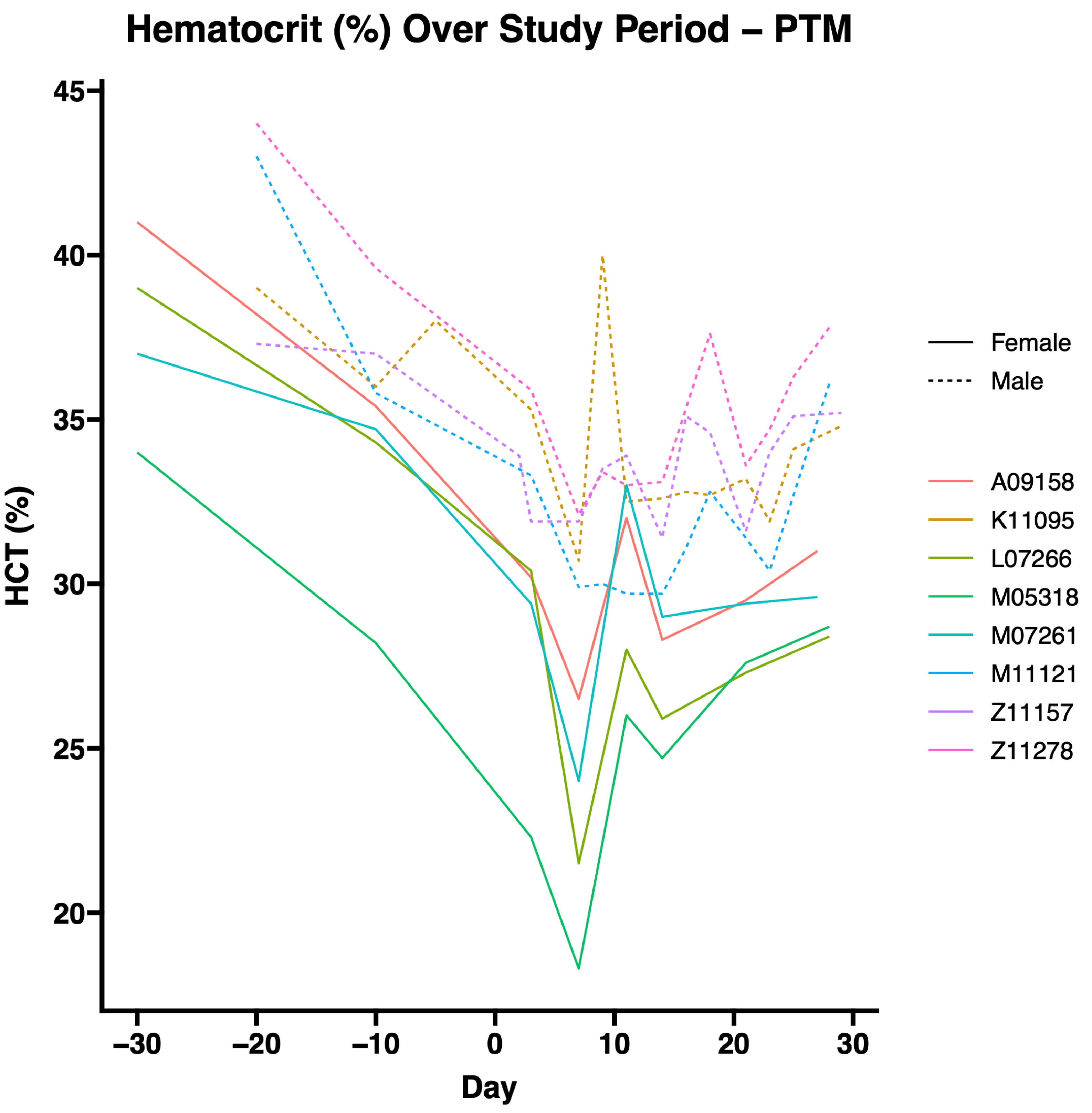
Hematocrit (%) Over Study Period – PTM
HCT (%)
Day
45
40
35
30
25
20
–30
–20
–10
0
10
20
30
Female
Male
A09158
K11095
L07266
M05318
M07261
M11121
Z11157
Z11278

Figure S2

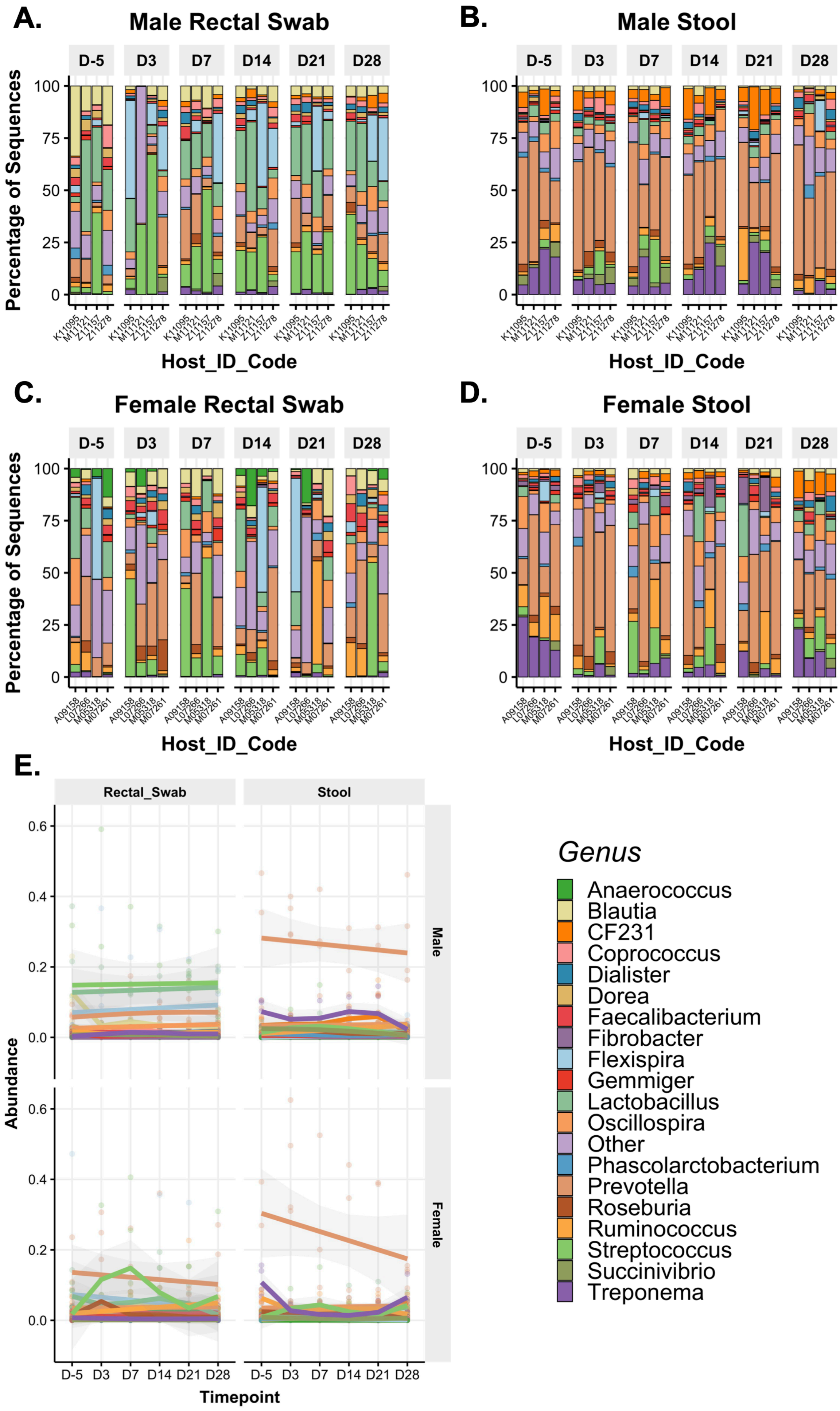
A.
Male Rectal Swab
B.
Male Stool
C.
Female Rectal Swab
D.
Female Stool
D-5
D3
D7
D14
D21
D28
Percentage of Sequences
Host_ID_Code
K11095
M11121
Z11157
Z11278
A09158
L07266
M05318
M07261
E.
Rectal_Swab
Stool
Male
Female
Abundance
Timepoint
Genus
Anaerococcus
Blautia
CF231
Coprococcus
Dialister
Dorea
Faecalibacterium
Fibrobacter
Flexispira
Gemmiger
Lactobacillus
Oscillospira
Other
Phascolarctobacterium
Prevotella
Roseburia
Ruminococcus
Streptococcus
Succinivibrio
Treponema

Figure S3

A.

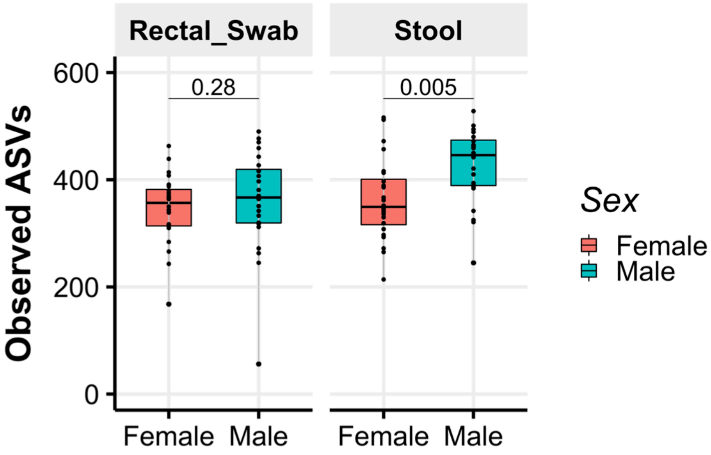
Rectal_Swab
Stool
600
400
200
0
0.28
0.005
Observed ASVs
Female
Male
Female
Male
Sex
Female
Male

Figure S4

A.

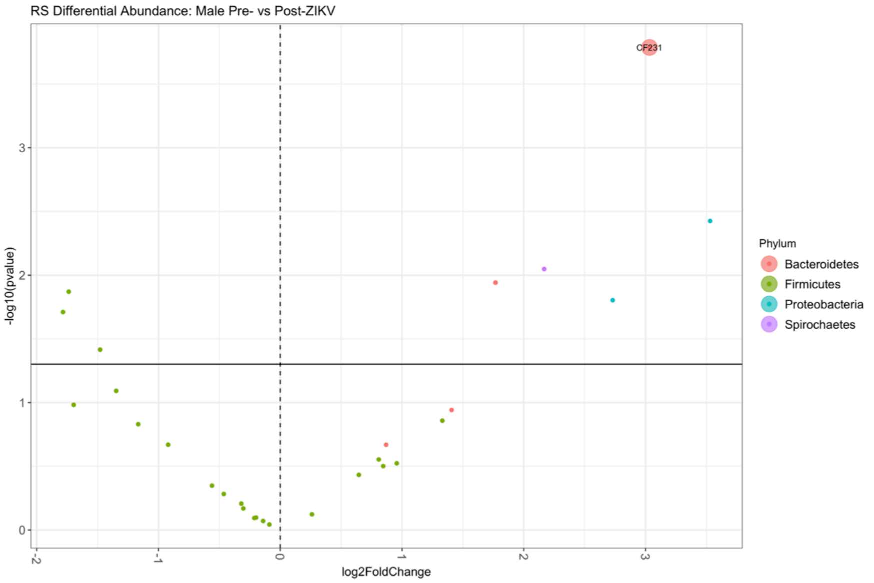

B.

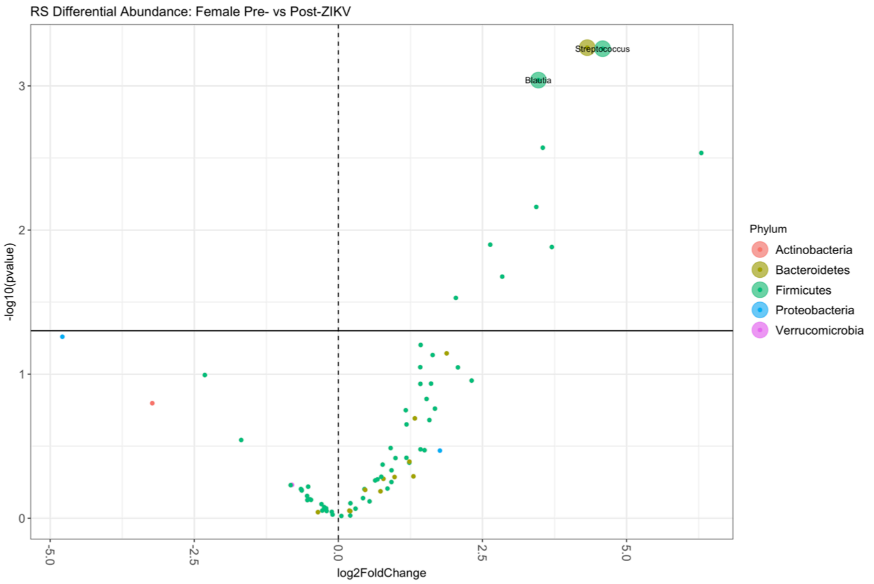

C.

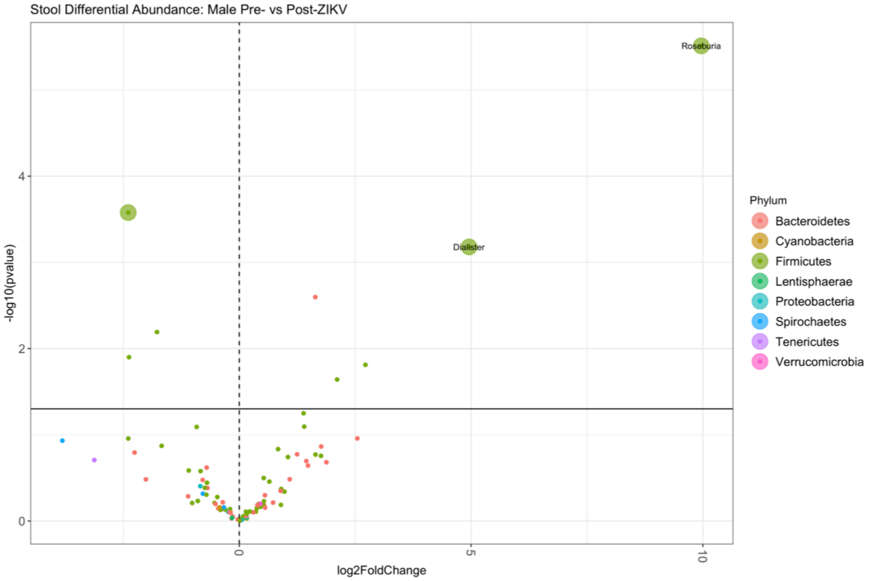

Table S1. Environmental variables for PTM

Animal ID	Species	Origin	Sex	Age	Run-Through, Paired Housing
M07261	PTM	WaNPRC at Tulane NPRC	F	8 yrs	NA
A09158	PTM	Yerkes NPC	F	8 yrs	NA
L07266	PTM	WaNPRC at Tulane NPRC	F	8.5 yrs	NA
M05318	PTM	WaNPRC at Tulane NPRC	F	10.5 yrs	NA
M11121	PTM	WaNPRC at SNBL TX	M	5.5 yrs	Z11278
Z11278	PTM	WaNPRC at SNBL TX	M	5.5 yrs	M11121
K11095	PTM	WaNPRC at SNBL TX	M	5.5 yrs	Z11157
Z11157	PTM	WaNPRC at SNBL TX	M	5.5 yrs	K11095

Potential environmental variables such as origin, sex, age and caging for the eight PTM.

Figure S1. Hematocrit is reduced in female PTM over the study period. Female PTM demonstrated a moderate then sharp decrease in the hematocrit (%) from -30 dpi to 7 dpi, followed by a general increase returning toward baseline. Different color lines represented different PTMs and day on the x-axis is relative to the days post infection. Line type reflects sex. Pre-infection days are represented by negative values.

Figure S2. ZIKV infection induced minimal shifts in bacterial community composition at the genus level in the rectum and stool of PTMs. 16s rRNA gene sequencing was used to characterize microbial genera in stool and rectal swabs collected from PTM prior to and throughout ZIKV infection. (A-D) Relative abundance taxonomic plots of microbial genera in male PTM rectal swabs (A), male PTM stool (B), female PTM rectal swabs (C) and female PTM stool (D). Vertical colored bars represent the percentage of total sequences for specific genera in individual animals prior to ZIKV infection and at each time point post-ZIKV infection. (E) Smoothed mean relative abundance of bacterial genera in each of the indicated sample types in male and female PTM. Solid colored lines represent the median abundance for specific bacterial genera. Grey shading overlaying each colored line represents standard error bounds. Matched colored dots surrounding each colored line represent the specific abundances of each bacterial genera for individual animals.

Figure S3. Increased richness in stool of male PTMs as compared to female PTMs. Bacterial community richness was assessed in rectal swabs and stool from female (pink) and male (teal) PTMs. Data from male and female PTMs at all time points were combined, regardless of ZIKV infection status. Box and whisker bars represent 25-75 percentile and minimum and maximum number of observed amplicon sequence variants. Horizontal bars within each box represent the median. Black dots that overlay box and whisker plots represent the total number of observed ASVs for individual animals at different time points. Statistical significance between the level of bacterial community richness in rectal swabs and stool of female and male PTMs was calculated using a Wilcoxon signed-rank test.

Figure S4. Alterations in the abundance of bacterial phyla following ZIKV infection. Shifts in bacterial ASVs away from baseline following ZIKV infection was assessed in stool and rectal swabs collected from male and female PTM prior to and throughout ZIKV infection. (A-C) Volcano plots depicting the log_2-fold change in the abundance of ASVs after ZIKV infection in male PTM rectal swabs (A), female PTM rectal swabs (B), and male PTM stool (C). Colored dots represent

individual bacterial ASVs. Large colored dots labeled with a genus indicate ASVs found to be significantly differentially abundant.
